# A pathogenic proteolysis–resistant huntingtin isoform induced by an antisense oligonucleotide maintains huntingtin function

**DOI:** 10.1172/jci.insight.154108

**Published:** 2022-09-08

**Authors:** Hyeongju Kim, Sophie Lenoir, Angela Helfricht, Taeyang Jung, Zhana K. Karneva, Yejin Lee, Wouter Beumer, Geert B. van der Horst, Herma Anthonijsz, Levi C.M. Buil, Frits van der Ham, Gerard J. Platenburg, Pasi Purhonen, Hans Hebert, Sandrine Humbert, Frédéric Saudou, Pontus Klein, Ji-Joon Song

**Affiliations:** 1 Department of Biological Sciences, KI for the BioCentury, Korea Advanced Institute of Science and Technology (KAIST), Daejeon, South Korea.; 2 University Grenoble Alpes, Inserm, U1216, CHU Grenoble Alpes, Grenoble Institut Neurosciences, Grenoble, France.; 3 ProQR Therapeutics NV, Leiden, Netherlands.; 4 Department of Biomedical Engineering and Health Systems, School of Engineering Sciences in Chemistry, Biotechnology and Health, KTH Royal Institute of Technology, Huddinge, Sweden.

**Keywords:** Neuroscience, Neurodegeneration

## Abstract

Huntington’s disease (HD) is a late-onset neurological disorder for which therapeutics are not available. Its key pathological mechanism involves the proteolysis of polyglutamine-expanded (polyQ-expanded) mutant huntingtin (mHTT), which generates N-terminal fragments containing polyQ, a key contributor to HD pathogenesis. Interestingly, a naturally occurring spliced form of *HTT* mRNA with truncated exon 12 encodes an HTT (HTT^Δ12^) with a deletion near the caspase-6 cleavage site. In this study, we used a multidisciplinary approach to characterize the therapeutic potential of targeting HTT exon 12. We show that HTT^Δ12^ was resistant to caspase-6 cleavage in both cell-free and tissue lysate assays. However, HTT^Δ12^ retained overall biochemical and structural properties similar to those of wt-HTT. We generated mice in which HTT exon 12 was truncated and found that the canonical exon 12 was dispensable for the main physiological functions of HTT, including embryonic development and intracellular trafficking. Finally, we pharmacologically induced HTT^Δ12^ using the antisense oligonucleotide (ASO) QRX-704. QRX-704 showed predictable pharmacology and efficient biodistribution. In addition, it was stable for several months and inhibited pathogenic proteolysis. Furthermore, QRX-704 treatments resulted in a reduction of HTT aggregation and an increase in dendritic spine count. Thus, ASO-induced HTT exon 12 splice switching from HTT may provide an alternative therapeutic strategy for HD.

## Graphical abstract

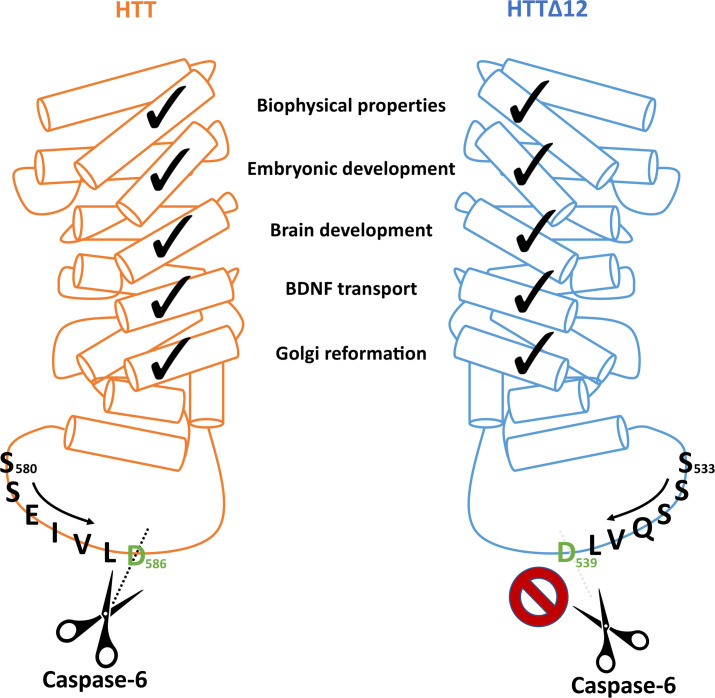

## Introduction

Huntington’s disease (HD) is caused by CAG trinucleotide repeat expansion in the *HTT* gene resulting in a polyglutamine-expanded (polyQ-expanded) mutant huntingtin (mHTT) protein at the N-terminus. The complex pathology of HD includes contributions of both toxic gain-of-function and loss-of-function mechanisms (1). Increasing evidence suggests that appropriate therapeutic strategies should maintain the function of the wt-HTT (wtHTT) protein. First, studies in animal models and patients with HD have indicated that changes in the levels of the wtHTT protein impact the progression of HD pathology (2, 3). Second, the function of wtHTT is crucial for brain development and maintenance, as illustrated by the developmental and degenerative phenotypes observed in conditional *Htt^KO^* mice (4, 5) and by the fact that patients with wtHTT hypomorphs show neurodevelopmental deficits (6).

Although the various cellular functions of HTT remain to be elucidated, several of the known functions highlight a crucial role for HTT in neuronal homeostasis (1). HTT promotes the synthesis and axonal transport of brain-derived neurotrophic factor (BDNF), resulting in a beneficial effect on striatal survival and the maintenance of the corticostriatal circuit (7, 8). In addition, by regulating the trafficking of protein complexes such as those in the pericentriolar material, HTT modulates cilia formation in various brain regions, including the ependymal layer of the intracerebral ventricles, thus controlling the circulation of cerebrospinal fluid and affecting brain homeostasis (7, 9).

Among the various events associated with HD pathogenesis, the proteolytic cleavage of mHTT is a key event, as it generates toxic N-terminal fragments that are hypothesized to be the main contributors to the disease. N-terminal fragments accumulate in large amounts in postmortem tissue from patients with HD (10, 11), and it has been established that the expression of the N-terminal fragments of mHTT induces markedly greater toxicity than the expression of full-length mHTT. A small N-terminal fragment as well as a larger C-terminal fragment are formed through a cascade of proteolytic cleavage, which is initiated at position Asp586 (D586) and mediated by caspase-6 or other caspases (12). The N-terminal HTT_1–586_ fragment is an unstable product that is further processed into smaller fragments (13). It has been demonstrated that D586 cleavage enables HTT to be accessed for proteolysis at upstream sites such as Arg167 (R167) (Cp2), which are normally inaccessible in the full-length protein due to the folded structure (11). The larger C-terminal fragment generated by D586 cleavage is also highly toxic (11). Importantly, D586 cleavage is required for YAC128 mouse models to acquire HD-like phenotypes (14). Moreover, wtHTT cleavage is thought to contribute to HTT loss-of-function pathology by reducing the amount of available full-length HTT protein, as evident from postmortem HD tissue analysis (11).

It has long been hypothesized that the generation of N-terminal fragments, such as the HTT_1-586_ fragment, is accelerated by a forward feedback loop in which cleavage products drive caspase activation, generating increased amounts of cleavage products (15). This hypothesis is supported by mouse genetic data, since caspase-6 hyperactivity is compensated by the genetic inactivation of the D586 site (16). A recent study provided a biochemical mechanism to explain the mechanism: the mHTT_1-586_ fragment specifically binds to pro–caspase-6 and stabilizes a conformation that leads to its activation (17). Caspase-6 was shown to present highly elevated activity in the postmortem HD striatum and cortex (16), further supporting this hypothesis. Elevated caspase-6 activity may contribute to the cleavage of a wide range of substrates in addition to HTT, which may have damaging effects on cellular proteomic homeostasis. Inactivating the D586 cleavage site has been hypothesized to prevent (i) the formation of the HTT_1–586_ fragment and subsequent cleavage of small toxic N-terminal fragments, (ii) the formation of a toxic C-terminal fragment, (iii) the loss of the protective full-length wtHTT, and (iv) caspase-6 hyperactivation. For these reasons, various attempts to prevent D586 cleavage have been tested, including treatment with caspase-6 inhibitors (18) and a D586-blocking Ab (19). While these approaches have shown some positive preclinical results, they have not yet been developed clinically.

Several splice variant HTT isoforms have been identified (20, 21). Among these isoforms, the HTT^Δ12^ isoform harbors a deletion of 135 nucleotides from exon 12, resulting in a 45 aa deletion. Interestingly, the deletion occurs in the proximity of the caspase-6 cleavage site (D586), raising the possibility that the HTT^Δ12^ isoform is resistant to caspase-6 cleavage. A previous study serendipitously discovered that an antisense oligonucleotide (ASO), originally designed for exon 12 and exon 13 skipping, shifts the canonical exon 12 splicing site by inactivating the canonical exon 12 splice donor site and activating an alternative site located 135 nucleotides upstream, which produces an HTT^Δ12^ isoform that is identical to the naturally occurring HTT^Δ12^ isoforms (20–22). Here, the ASO used by Evers and colleagues was modified with 2′-O-methoxyethyl (MOE) phosphorothioate chemistry to generate a compound named QRX-704, which is a preclinical therapeutic candidate for the treatment of HD (22).

Given the clinical risks associated with targeting both the wt and mHTT alleles and the adverse phenotypes observed in mice and patients with reduced wtHTT levels, we investigated whether HTT^Δ12^ maintains the functions of canonical HTT, which is a prerequisite for the possible therapeutic application of QRX-704. Our biophysiochemical analysis of HTT^Δ12^ revealed that HTT^Δ12^ is resistant to cleavage by caspase-6 and that HTT^Δ12^ maintains biochemical and structural properties similar to those of canonical HTT. We further generated an HTT^Δ12^ isoform knock-in mouse model and showed that the HTT^Δ12^ isoform supports normal embryonic development and maintains the functionality of HTT in intracellular trafficking activities. Finally, we pharmacologically induced exon 12 splicing-switching in an HD mouse model using QRX-704. We observed that the HTT^Δ12^ isoform supported the normal function of wtHTT in mice and that the pathogenic proteolysis was significantly reduced, leading to a reduction in HTT aggregation and an increase in the dendritic spine count.

Our data show that ASO-induced HTT^Δ12^, which is resistant to caspase-6 cleavage, can perform the functions of full-length HTT, providing a potentially novel strategy for modulating the pathology of HD.

## Results

### Recombinant HTT^Δ12^ is resistant to caspase-6 cleavage.

**Figure 1:**
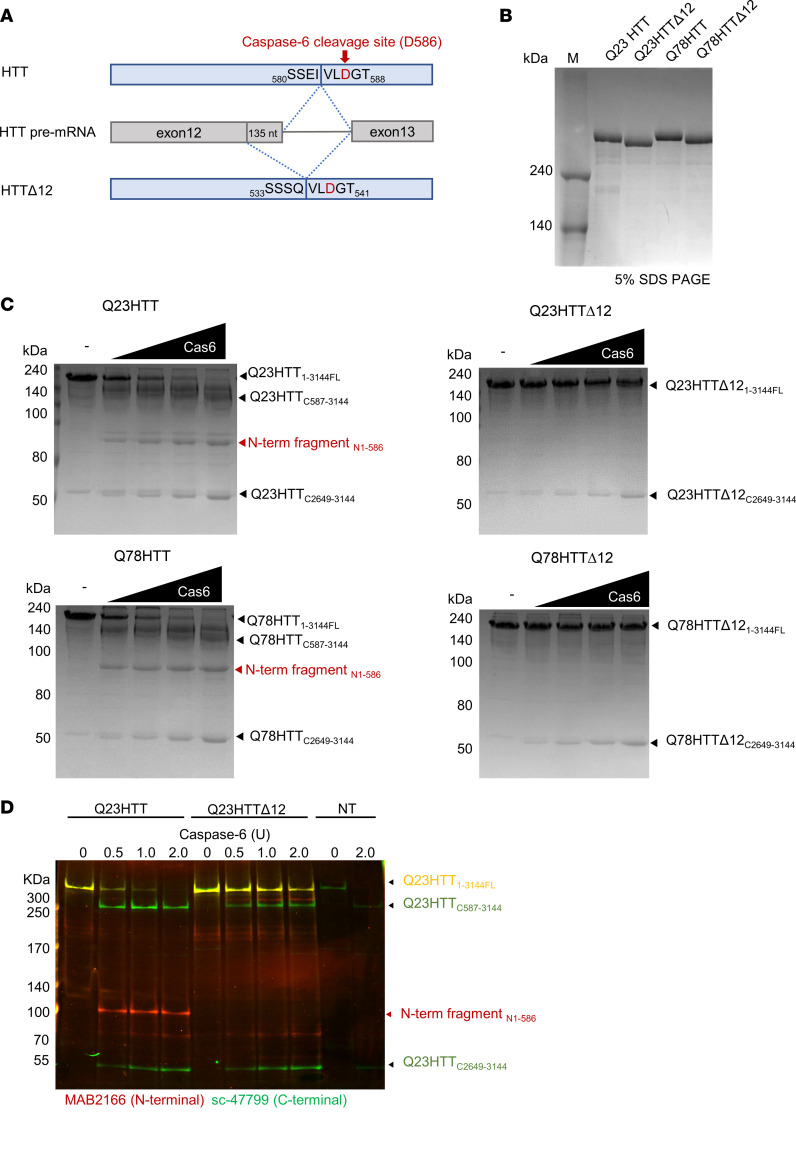
HTT^Δ12^ is resistant to caspase-6 cleavage. (**A**) A schematic of the alternative splicing to generate the HTT^Δ12^ isoform. The alternative splicing leads to the deletion of a part of the caspase-6 site. The red arrow indicates the caspase-6 cleavage site. (**B**) Highly purified full-length recombinant HTTs (Q23HTT, Q23HTT^Δ12^, Q78HTT, and Q78HTT^Δ12^). (**C**) Caspase-6 cleavage assay. The red arrows indicate the N-terminal fragment (N-term fragment_N1-586_) generated by caspase-6 (Cas6) cleavage. (**D**) Caspase-6 cleavage assay of COS7 cell lysates transfected with either Q23HTT or Q23HTT^Δ12^ or nontransfected (NT) and treated with recombinant Caspase-6 in indicated Us for 5 hours, followed by Western blot detection with MAB2166 (red, N-terminal) or sc-47799 (green, C-terminal) Abs (overlapping signal yellow).

An alternatively spliced form of *HTT* mRNA in which 135 nucleotides from the 3′ end of exon 12 are spliced out has been reported as a naturally occurring isoform in human cell lines (20). This alternative splicing results in the deletion of the region from 539 to 583 aa in HTT, which is close to the caspase-6 cleavage site at position D586 (_583_IVLD_586_) (Figure 1A) (23). This truncated HTT isoform (HTT^Δ12^) is likely to be resistant to cleavage by caspase-6 as its recognition site has been modified (QVLD). To test the caspase resistance of HTT^Δ12^, we first generated recombinant full-length HTT proteins containing either 23 polyQ repeats (Q23HTT, wtHTT) or 78 polyQ repeats (Q78HTT, mHTT) and corresponding HTT^Δ12^ proteins (Q23HTT^Δ12^ and Q78HTT^Δ12^) using the previously established baculovirus-mediated insect cell expression system for producing full-length HTT with high homogeneity (Figure 1B) (24, 25). We next incubated HTT proteins (Q23HTT, Q78HTT, Q23HTT^Δ12^, and Q78HTT^Δ12^) with increasing amounts of caspase-6 and examined their proteolysis. Caspase-6–induced proteolysis of Q23HTT and Q78HTT led to the generation of proteolytic products with the expected size of 90–100 kDa that corresponds to the N-terminal fragment (N-term fragment_N1-586_) cleaved by caspase-6, while the full-length HTT proteins (Q23HTT_1-3144FL_) disappeared (Figure 1C). However, we did not observe the corresponding bands when the Q23HTT^Δ12^ and Q78HTT^Δ12^ proteins were incubated with recombinant caspase-6 (Figure 1C), indicating that HTT^Δ12^ proteins are resistant to caspase-6 cleavage. Interestingly, we noticed the presence of an additional fragment (~50 kDa) that was generated upon caspase-6 treatment regardless of the presence or absence of exon 12, suggesting the existence of an additional caspase-6–sensitive site (Figure 1C and Supplemental Figure 1A; supplemental material available online with this article; https://doi.org/10.1172/jci.insight.154108DS1). To identify this potential second site, we subjected the bands located near the 50 kDa position to N-terminal sequencing by Edman degradation. HPLC chromatographs generated during the N-terminal sequencing analysis showed that fragments generated from both the Q23HTT and Q23HTT^Δ12^ proteins start with the same Ala-Pro-Ala sequence, which corresponds to aa 2,649 (Supplemental Figure 1, B and C). The sequence upstream of this Ala-Pro-Ala sequence is Glu-Glu-Glu-Ala-Asp (EEEAD_2648_), suggesting that caspase-6 cleaves HTT immediately after D2648 (Supplemental Figure 1D). Interestingly, the EEEAD sequence has also been found to be a caspase-6 cleavage site within DNA topoisomerase I (26). However, whether the second caspase-6 cleavage site plays a role in HD pathology remains to be investigated.

We next asked whether the HTT^Δ12^ isoform generated in cells is also resistant to caspase-6 cleavage. We transfected Q23HTT or Q23HTT^Δ12^ into COS7 cells and incubated the cell lysates with recombinant caspase-6. Full-length HTTs and HTT fragments were detected with the MAB2166 and sc-47799 Abs targeting the N-terminus and C-terminus of HTT, respectively (Figure 1D). Consistent with the assay based on recombinant HTT^Δ12^, the HTT^Δ12^ expressed in cells was also found to be resistant to caspase-6 cleavage in the N-terminal region. These data show that both the Q23HTT^Δ12^ and Q78HTT^Δ12^ proteins are resistant to caspase-6 cleavage at position D586.

### The truncation of exon 12 does not modify the biochemical or structural properties of HTT.

**Figure 2:**
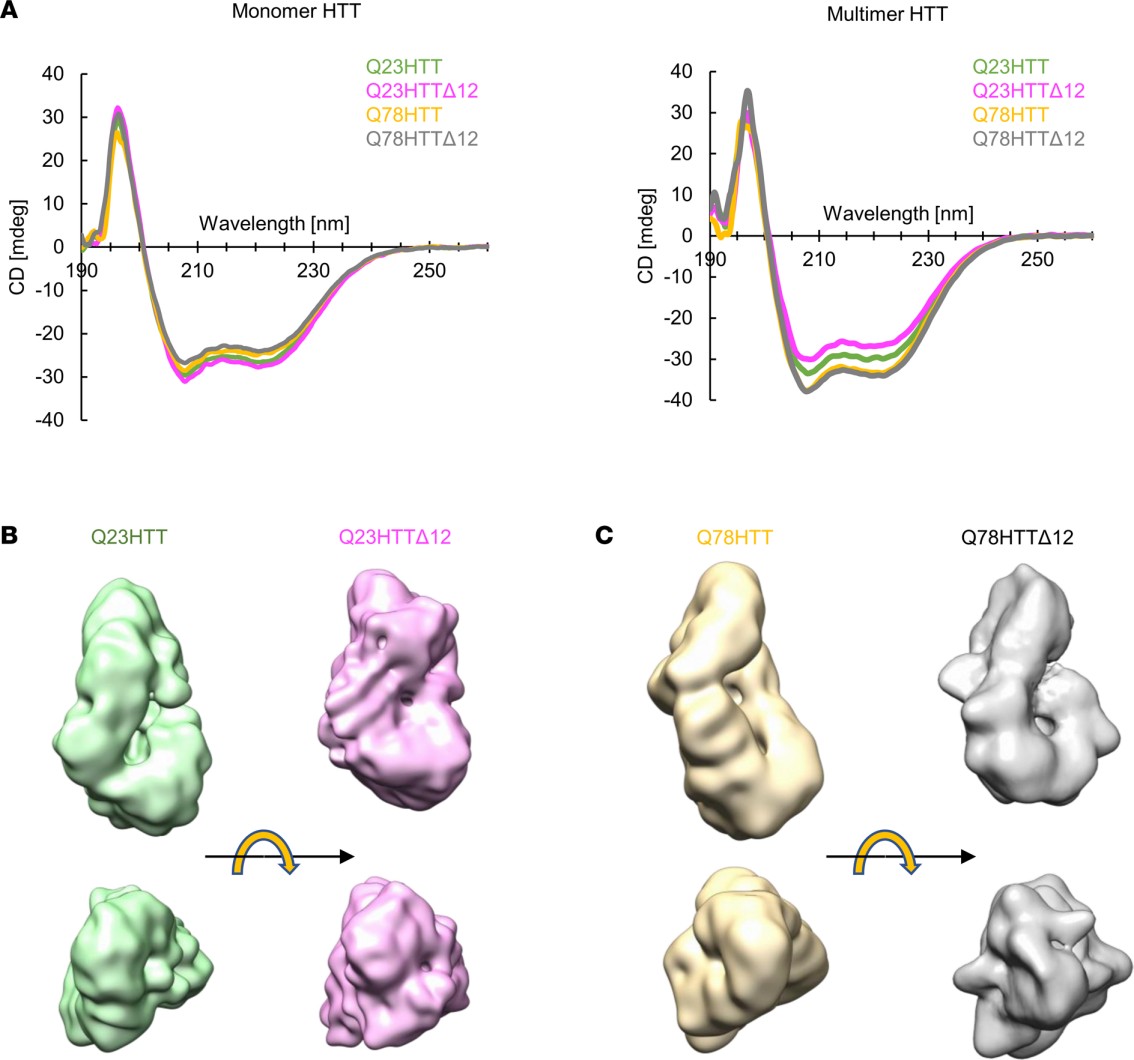
Comparative biophysical analysis of HTT and HTT^Δ12^ proteins. (**A**) Circular dichroism (CD) spectrum of multimer HTTs and monomer HTTs between 190 and 260 nm wavelengths. HTT and HTT^Δ12^ proteins show similar patterns of CD spectra. (**B** and **C**) Comparative cryo-EM analysis on HTT and HTT^Δ12^ proteins. The cryo-EM maps of HTTs (Q23HTT in green and Q78HTT in yellow) are from our previous work (28). The cryo-EM maps of HTT^Δ12^ proteins (Q23HTT^Δ12^ in pink and Q78HTT^Δ12^ in gray) are from this study.

Alternative splicing skipping a portion of exon 12 results in a deletion of 45 aa in the HTT protein (HTT^Δ12^). Hence, we asked whether HTT^Δ12^ retains the biochemical and structural properties of HTT. To address this question, we characterized the structures of Q23HTT^Δ12^ and Q78HTT^Δ12^. First, to examine whether the structural integrity of HTT is maintained in the Q23HTT^Δ12^ and Q78HTT^Δ12^ proteins, we utilized circular dichroism (CD), which enables the examination of secondary structures. We found that both HTT and HTT^Δ12^ (either with the Q23 and Q78) naturally form oligomers (Supplemental Figure 2A). As purified monomeric HTT undergoes multimerization, we crosslinked HTT with disuccinimidyl suberate (DSS), and the stable monomeric HTT proteins were separated via sucrose gradient ultracentrifugation (Supplemental Figure 2, B and C). We then measured the structural properties of the monomeric HTT proteins and HTT in multimer form using CD (Figure 2A). We measured the CD spectra for the different HTT proteins and observed the lowest absorption at wavelengths of approximately 210 nm and 220 nm, which is consistent with the HTT composition, consisting of helices that form HEAT repeats (27). We found that the CD spectra obtained for HTT and HTT^Δ12^ were similar regardless of the polyQ length. We concluded that the truncation of exon 12 did not affect the structure of the HTT protein at the secondary structure level.

To verify that the exon 12 truncation does not alter the HTT structure, we analyzed the structures of the Q23HTT^Δ12^ and Q78HTT^Δ12^ mutants using cryo-electron microscopy (cryo-EM) and compared the structures with those structures of HTT proteins that we had previously resolved (28). We vitrified Q23HTT^Δ12^ and Q78HTT^Δ12^ and collected cryo-EM micrographs (Supplemental Figures 3 and 4). We then processed the micrographs and obtained low-resolution maps of Q23HTT^Δ12^ and Q78HTT^Δ12^. Despite the low resolution, we were able to conclude that the overall structures of both Q23HTT^Δ12^ and Q78HTT^Δ12^ were similar to those of the HTT proteins (Figure 2, B and C, and Supplemental Figure 5). These comparative biophysical analyses show that HTT^Δ12^ proteins maintain structural properties similar to those of the HTT protein.

### The HTT^Δ12^ isoform is sufficient to support mouse embryonic development.

**Figure 3:**
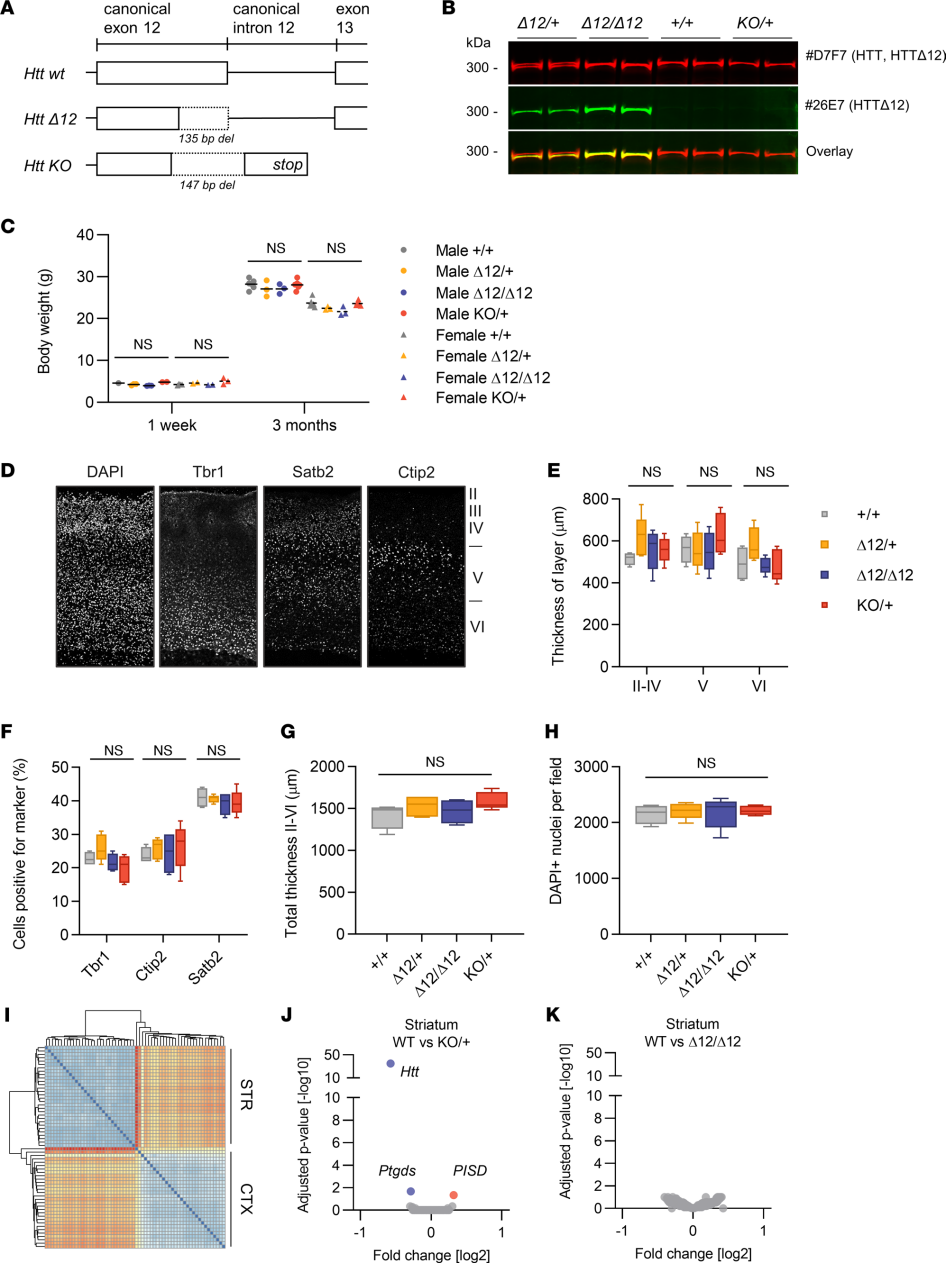
HTT^Δ12^ isoform supports mouse embryonic development. (**A**) Schematic of mouse *Htt*^+/+^ (wild-type), *Htt^Δ12^*, and *Htt^KO^* *exon12* alleles. (**B**) Western blot of HTT isoform expression in brains of *Htt^Δ12^*, *Htt^KO^*, and *wt* littermates using the D7F7 Ab (canonical HTT) and 26E7 Ab (HTT^Δ12^ isoform). (**C**) Body weights of 1- and 3-month-old male and female Htt^+/+^, Htt^Δ12/+^, Htt^Δ12/Δ12^, and Htt^KO/+^ mice, *n* = 3–5 mice per group. (**D**) Representative images of individual immunofluorescence stainings of cortical sections with indicated markers. (**E**) Thickness of cortical layer II–IV, V, and VI was measured from confocal images of brain sections from P7 from HTT^+/+^, HTT^Δ12/+^, HTT^Δ12/Δ12^, and HTT^KO/+^ mice. Thickness in micrometers calculated from the average layer thickness of the left and right hemisphere from 4 HTT^+/+^ or 5-HTT^Δ12/+^, 5 HTT^Δ12/Δ12^, and 5 HTT^KO/+^ mice. (**F**) Average numbers of Tbr1-, Ctip2-, and Satb2-positive cells compared with the total number of nuclei. (**G**) Averages of the total thickness of cortical layers II–VI of the right and left hemispheres in μm. (**H**) Total number of nuclei within the cortical layers was determined from the DAPI signal for each genotype. The average number from both hemispheres was averaged for the 4–5 littermates. The box plots depict the minimum and maximum values (whiskers), the upper and lower quartiles, and the median. The length of the box represents the interquartile range. (**I**) Hierarchical clustering of relative sample distance of transcriptome analysis of adult HTT^Δ12^ brain (HTT^+/+^, HTT^Δ12/+^, and HTT^Δ12/Δ12^) versus HTT^KO^ (HTT^+/+^ and HTT^KO/+^) (GSE209893). Littermates at the age of 3 months were compared and cortex and striatum were analyzed by RNA-Seq (*n* = 3 males + 3 females per group). (**J**) Volcano plot of differentially expressed genes showing Benjamini-Hochberg–adjusted *P* values and fold-change in striata of Htt^+/+^ versus Htt^KO/+^ and (**K**) Htt^+/+^ versus Htt^Δ12/Δ12^ mice. Statistics: 1- or 2-way ANOVA with Tukey’s multiple comparison test.

The mouse *Htt* gene is critically required for mouse embryonic development, as illustrated by early embryonic lethality at embryonic day 7.5 in homozygous null mice (29). To investigate whether the truncation of exon 12 may affect the critical function of wtHTT during embryonic development, we used a CRISPR/Cas9 homology-directed repair (HDR) knock-in strategy and generated a 135 bp deletion at the 5′ end of the endogenous mouse *Htt* gene in C57BL/6 wt mouse embryonic stem cells, producing an *Htt*^Δ12^ allele containing a new splice donor site that mimics the alternatively spliced human isoform (Figure 3A). A founder line, in which HDR was unsuccessful, contained an imprecise 147-bp deletion leading to the removal of the normal splice donor and this truncated transcript was used as a control KO allele (*Htt^KO^*). Breeding heterozygous *Htt*^Δ12^ or *Htt^KO^* to create homozygosity among offspring led to the birth of viable *Htt*^Δ*12/Δ*12^ pups at the expected frequencies (Table 1). However, no *Htt^KO/KO^* animals were viable (Table 1), indicating that the *Htt*^Δ12^ allele can functionally substitute for the wt allele during embryonic development. A Western blot analysis of adult brain tissue verified normal expression levels of the HTT^Δ12^ protein, which was detected using a monoclonal Ab raised specifically against the newly formed Δ12 epitope (Figure 3B). The animals displayed no apparent morphological or behavioral abnormalities and remained fertile. The mutant mice showed normal body weights (Figure 3C) and survived for at least 18 months.

HTT has important functions in brain development, notably in cortical development. Both conditional loss-of-function and expanded CAG models of young postnatal animals showed a defect in cortical layering (30–33). We immunostained P7 cortical sections to detect the presence of Tbr1, Ctip2, and Satb2, which are neuronal markers of layer VI neurons, layer V neurons, and callosal projecting neurons (mainly expressed in upper-layer II/III/IV neurons). Using these markers, we measured the thickness of the cortical layers, the number of cells showing positive for specific cortical markers, and the total cortical thickness and cell density (Figure 3, D–H). No differences were found between wt and homozygous *Htt*^Δ12^ within precision limits of the measurements, supporting the presumption that *Htt*^Δ12^ mice undergo normal cortical development. Finally, a transcriptome analysis of cortical and striatal tissue in 3-month-old mice based on RNA-Seq did not reveal altered expression patterns or differentially expressed genes (Figure 3, I–K).

Taken together, the results indicate that the *Htt*^Δ12^ allele supports normal embryonic development and HTT functionality in the central nervous system of adult mice.

### The truncation of exon 12 does not alter the cytoskeletal function of full-length HTT.

**Figure 4:**
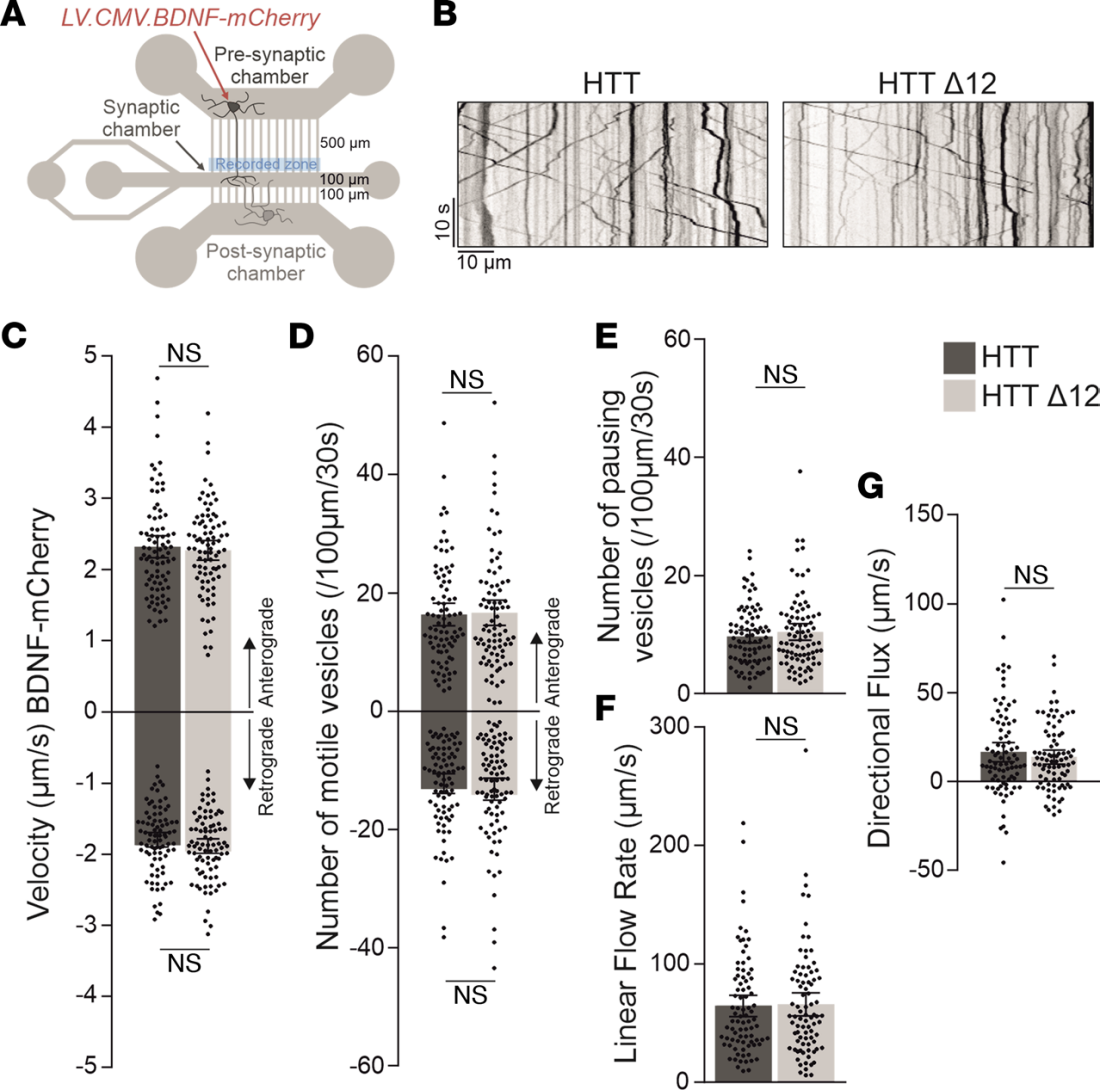
HTT^Δ12^ does not modify the transport of BDNF vesicles. (**A**) Schematic representation of the microfluidic device used to reconstruct cortico-cortical network with indication of the video recording zone for BDNF-mCherry–containing vesicles. The device contains 3 compartments: the presynaptic, the postsynaptic, and the synaptic compartments, which are connected by microchannels. (**B**) Representative kymographs showing BDNF-mCherry axonal trafficking in HTT and HTT^Δ12^ neurons. (**C**) Anterograde and retrograde segmental velocities of BDNF-mCherry vesicles. (**D**) Number of anterograde and retrograde vesicles trafficking along 100 μm of axon. (**E**) Number of pausing vesicles along 100 μm of axon. (**F**) Linear flow rate (μm/s). (**G**) Directional flux (μm/s). The number of axons per condition in at least 2 independent experiments (Supplemental Data 1); *n* = 84 wt and *n* = 84 *Htt^Δ12/Δ12^*. D’Agostino-Pearson normality test followed by Mann-Whitney test.

To determine whether the truncation of exon 12 alters HTT function, we focused on functional assays measuring well-established wtHTT functions. These assays are mostly related to HTT cytoskeletal functions and include the evaluation of BDNF axonal transport, Golgi apparatus reformation after microtubule (MT) disruption, and ciliogenesis (1). HTT regulates the axonal transport of BDNF through an MT- and molecular motor-dependent mechanism (7). A reduction in HTT levels increases the percentage of static vesicles and reduces their velocities while increasing their pausing time (7). To examine whether the truncation of exon 12 in HTT influences the transport of BDNF-containing vesicles in vitro, we reconstructed cortico-cortical neuronal networks on silicone-based microchips (8, 34) (Figure 4A). We plated cortical neurons from E15.5 wt and *Htt*^Δ*12/Δ*12^ homozygous knockin mouse embryos in the pre- and postsynaptic compartments of microfluidic devices (Figure 4A). We waited 3 hours after plating and then transduced cortical neurons in the presynaptic compartment with a lentivirus-expressing BDNF tagged with mCherry. After 12 days in vitro, the trafficking of BDNF-mCherry–containing vesicles in the distal part of the microchannels containing the presynaptic cortical axons was measured via spinning confocal videomicroscopy. At this stage, the cortico-cortical neurons were fully connected, similar to other neuronal circuits (34, 35). We generated kymographs on the basis of the obtained videos (Figure 4B) and quantified the segmental velocity (the speed of a given vesicle without pauses), the number of BDNF-moving vesicles traveling in the anterograde and retrograde directions, and the number of BDNF-pausing vesicles (Figure 4, C–E). We found no significant difference in the anterograde or retrograde velocities (Figure 4C), the number of vesicles moving in the anterograde or retrograde direction (Figure 4D), or the number of paused vesicles (Figure 4E) between cortical neurons from wt and *Hdh*^Δ*12/Δ*12^ homozygous mice (Mann-Whitney test). We also found no difference in the linear flow accounting for the number of vesicles and their overall velocity (Figure 4F) or in the net directional flux accounting for the direction of their movement compared with the soma and axon terminus (Figure 4G). These data indicate that the truncation of exon 12 in HTT does not influence the trafficking of vesicles along cortical axons.

**Figure 5:**
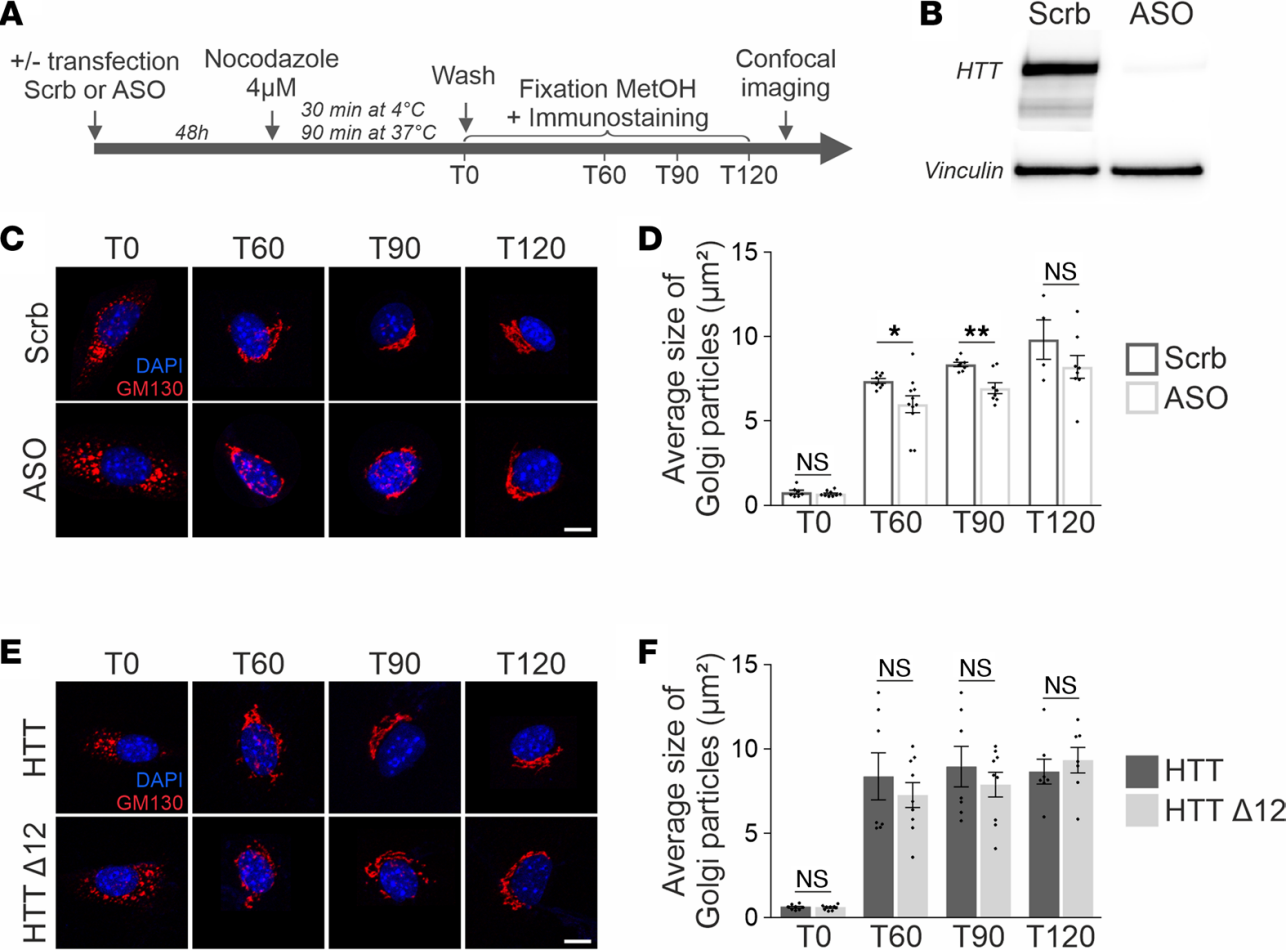
HTT^Δ12^ does not modify Golgi reformation. (**A**) A schematic description of the protocol used for transfection and Golgi reformation assays. (**B**) Fibroblasts were transfected with ASO or Scrb and analyzed by Western blot using the 4C8 anti-HTT. Vinculin is used as a protein-loading control. (**C**) Representative images of Golgi reformation in fibroblasts cells treated with ASO or Scrb at T0, T60, T90, and T120 after microtubule depolymerization. (**D**) Golgi reassembly is measured as the average size of Golgi particles (μm^2^) at the indicated time points after nocodazole washout in ASO or Scrb conditions. Results were obtained from 3–5 independent experiments in which 4–11 different cultures were analyzed. All comparisons are ASO versus Scrb at different times after nocodazole washout. Shapiro-Wilk normality test followed by 2-tailed unpaired Student’s *t* test: **P < 0.01; *P < 0.05. Scale bar: 10 μm. (**E**) Representative images of Golgi reassembly in fibroblasts expressing HTT or HTT^Δ12^ at different times after nocodazole washout. (**F**) Golgi reassembly is measured as in **D**. Results were obtained from 3–4 independent experiments in which 7–9 cultures were analyzed (Supplemental Data 1). All comparisons are HTT versus HTT^Δ12^ at different times after nocodazole washout. D’Agostino-Pearson normality test followed by Mann-Whitney test. Scale bar: 10 μm.

HTT ensures the maintenance of the structure of the Golgi apparatus through a mechanism that involves HTT interaction with the dynein/dynactin complex and the MT network (36–38). We investigated whether the truncation of exon 12 in HTT influences HTT function in the MT-dependent assembly of the Golgi apparatus using a Golgi reformation assay (Figure 5A). For this assay, primary cultures of fibroblasts obtained from E13–E15 mouse embryos were cultured and treated with nocodazole (4 μM) to allow complete MT depolymerization and Golgi dispersion. Golgi reassembly was monitored at several time points after nocodazole washout (T0, T60, T90, and T120) by measuring the average size of Golgi particles stained with GM130, a marker of cis-Golgi, as previously reported (37). We first validated our assay by evaluating the effect of *Htt* downregulation using an ASO (Figure 5B). In control cells that were treated with a scrambled oligonucleotide (Scrb), the Golgi structure became centrally reorganized after nocodazole washout, concomitant with the reformation of the MT network. In contrast, as previously reported (37), the silencing of *Htt* was associated with a delay in Golgi reformation, initiated 60 and 90 minutes after nocodazole washout (Figure 5, C and D) (Scrb vs. ASO at each time point; Student’s *t* test: ns, *P < 0.05, and **P < 0.01). We next performed a Golgi reformation assay with fibroblasts derived from wt and *Htt*^Δ*12/Δ*12^ homozygous knockin mouse embryos. The kinetics of Golgi reformation were similar under these 2 conditions (Figure 5, E and F) (HTT vs. HTT^Δ12^ for each time point; Mann-Whitney *U* test). Thus, these data indicate that the truncation of exon 12 has no significant effect on Golgi reformation, which is a mechanism that involves MTs and the dynein retrograde motor.

**Figure 6:**
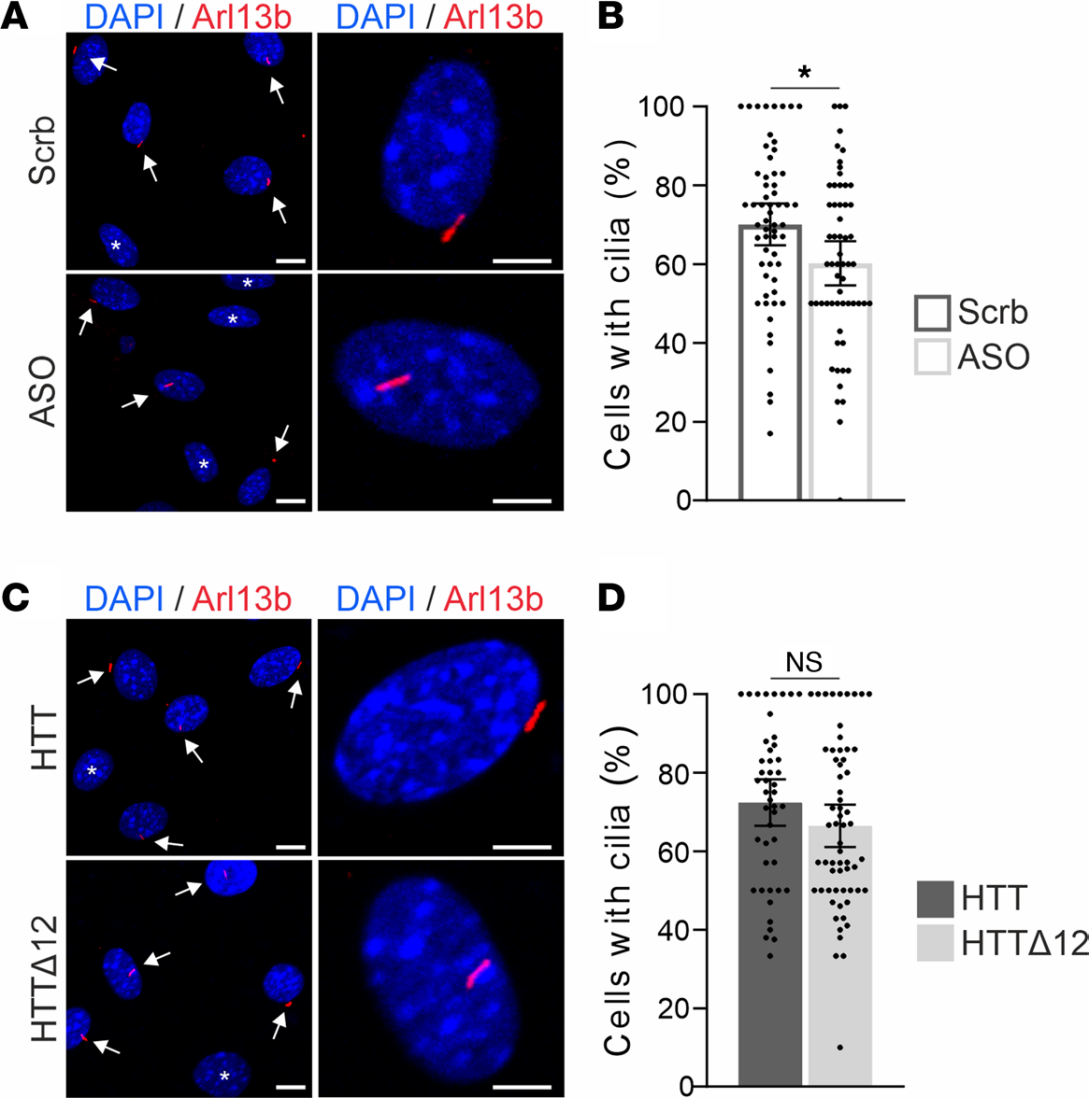
HTT^Δ12^ does not influence cilia assembly. (**A**) Representative images of primary cilia (Arl13b immunostaining) induced by serum starvation in fibroblasts transfected with ASO or Scrb. (Arrows indicate cells with primary cilia and stars indicate cells without cilia. Scale bars: 10 μm and 5 μm for higher magnification.) (**B**) Bar graphs show the percentage of cells with primary cilia in control (Scrb) or HTT-depleted (ASO) fibroblasts. Results were obtained from 7 cultures from 4 independent experiments in which *n* = 59 regions of interest (ROI) were analyzed. D’Agostino-Pearson normality test followed by unpaired 2-tailed Student’s *t* test: *P < 0.05. (**C**) Representative images of primary cilia (Arl13b immunostaining) of HTT or HTT^Δ12^ fibroblasts. (Arrows indicate cells with primary cilia and stars indicate cells without cilia. Scale bars: 10 μm and 5 μm for higher magnification.) (**D**) Bar graphs show the percentage of cells with primary cilia in HTT and HTT^Δ12^ fibroblasts. Results were obtained from 6–8 cultures from 3 independent experiments in which *n* = 46 HTT and *n* = 62 HTT^Δ12^ ROI were analyzed (Supplemental Data 1). D’Agostino-Pearson normality test followed by unpaired 2-tailed Student’s *t* test.

Finally, we investigated another cytoskeletal function of HTT: ciliogenesis. HTT is localized to the centrosome through MT-dependent transport, where it forms a complex with the pericentriolar material 1 (PCM1) protein and HTT-associated protein 1 (9). HTT depletion leads to the dispersion of PCM1 satellites from centrosomes and impairs primary cilia formation, as illustrated by a decrease in cells with cilia when HTT levels are reduced (9). We transfected fibroblasts with ASO or Scrb for 24 hours and then serum-starved the cells for 48 hours to induce primary cilia formation. We immunostained fibroblasts with the cilia marker Arl13b (Figure 6, A and C) and quantified the percentage of cells with primary cilia. We found that reducing HTT levels affected the percentage of cells with primary cilia (Figure 6B) (Student’s *t* test, *P < 0.05). Importantly, the truncation of exon 12 did not influence the percentage of fibroblasts with cilia (Figure 6D) (Student’s *t* test). Together, these results indicate that the truncation of exon 12 may have no significant effect on cilia formation.

### Generating the HTT^Δ12^ isoform via a pharmacological ASO approach.

**Figure 7:**
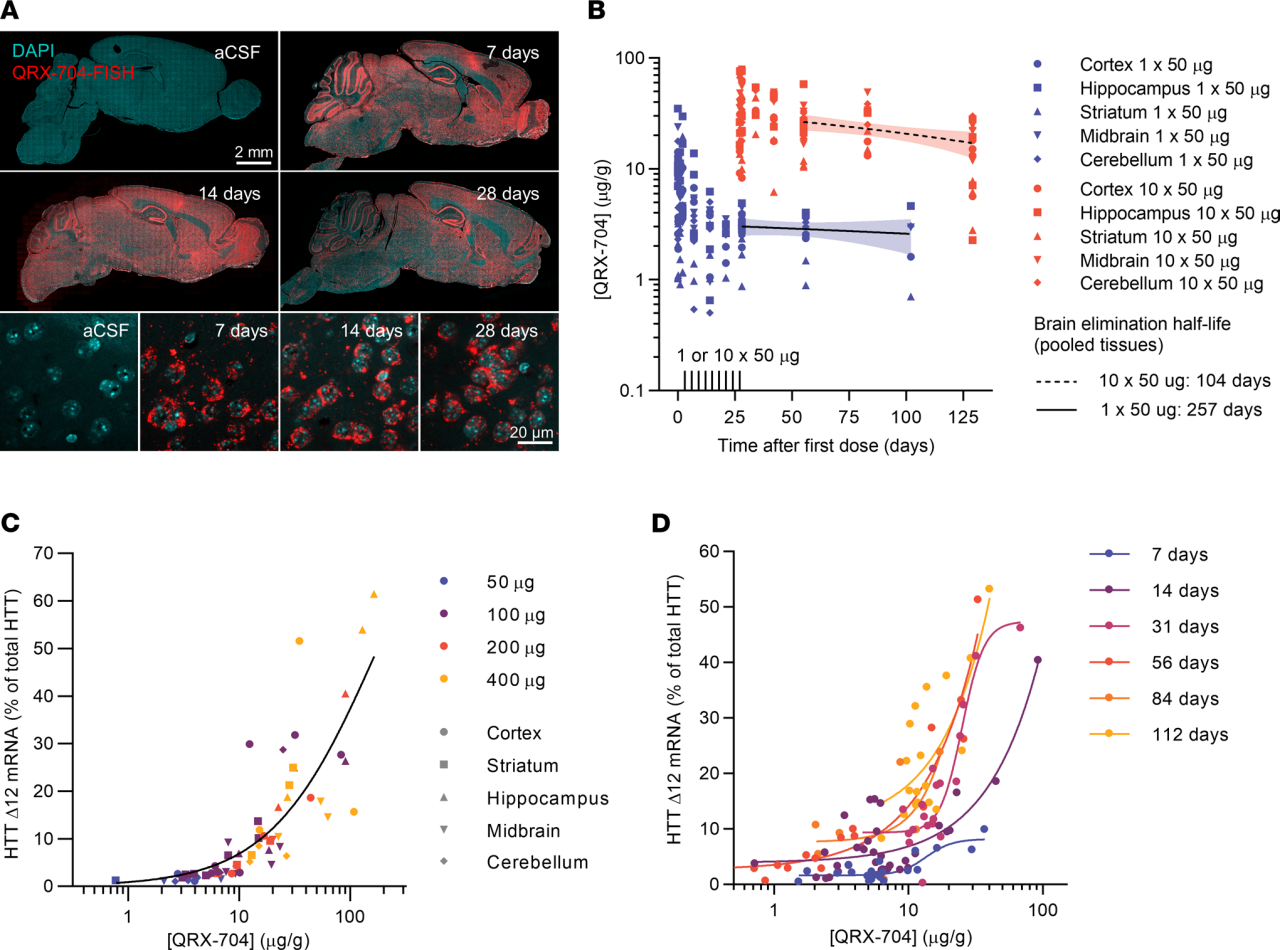
Biodistribution and pharmacology of HTT^Δ12^-activating oligonucleotide QRX-704. (**A**) Biodistribution of QRX-704 or artificial CSF (aCSF; vehicle) administered by a single i.c.v. bolus injection (500 μg) in wt mice, analyzed at 7, 14, or 28 days by FISH. Upper panels show tissue distribution in sagittal sections of mouse brain, lower panels are high magnification images of striatum showing cellular uptake. Scale bars: upper panels 2 mm, lower panels 20 μm. (**B**) QRX-704 PKs after either a single dose of 50 μg (blue) or 10 doses in 3-day intervals (red) in brain tissues as depicted, collected at time points between 1 hour and 102 days after last dose. Terminal elimination half-life was calculated by linear regression between the last 3 sample points on pool of tissues, displayed with 95% CIs. (**C**) Single-dose (50–400 μg) PK-PD 2 weeks after dosing, depicting HTT^Δ12^ mRNA as percentage of total HTT mRNA in indicated brain tissues sampled from the right hemisphere, and corresponding tissue samples of the left hemisphere analyzed for QRX-704 concentration, 4-point logistic regression of pooled samples. (**D**) Single-dose (200 μg) PK-PD analyzed 7–112 days after dosing, analyzed as in **C**. *n* = 4 animals per group and time point for all panels; however, several PK data points are unavailable due to samples not meeting bioanalysis acceptance criteria.

The exon 12 alternative splice donor site responsible for producing the HTT^Δ12^ isoform is naturally active at low levels in at least some cell types (20). A previously developed ASO that serendipitously activates this splice site was used to enable partial in-frame exon 12 skipping (22). This mechanism is also described as isoform switching. We optimized the ASO developed by Evers et al. (22) into an MOE compound with phosphorothioate linkages and designated it as QRX-704. A single intracerebroventricular bolus of QRX-704 (500 μg) was injected in wt C57BL6/J mice, and FISH was performed. The results showed a widespread and even distribution of stained QRX-704 throughout the brain and detectable uptake in nearly all cells (Figure 7A). The distribution pattern at the tissue and cellular levels did not change markedly between the time points of the study up to 28 days, as expected from the stabilizing ASO chemistry. The QRX-704 ASO was well tolerated and did not cause any apparent astrocytosis compared with the vehicle treatment (Supplemental Figure 6).

We performed a pharmacokinetic (PK) analysis with wt FVB mice administered either a single 50 μg dose or 10 repeated 50 μg doses in 3-day intervals. Tissues were collected at time points ranging from 1 hour to 102 days after the last treatment and analyzed for the tissue drug concentration by hybridization ELISA. The results obtained from pooled brain tissues indicated dose-proportional concentrations after initial clearance and a single-dose mean elimination half-life of 257 days (Figure 7B), similar to other fully MOE-modified ASOs (39). Since the mouse *Htt* gene does not contain the alternative splice site, we employed an FVB-YAC128 transgenic model carrying a full human HTT transgene for pharmacodynamic (PD) studies. We administered single doses of 50–400 μg and performed PK and PD analyses on *HTT* mRNA after 14 days. We observed dose-response splice switching to the HTT^Δ12^ isoform in all the regions investigated without reaching an RNA-level efficacy plateau (Figure 7C). To assess the PD time course, we administered a single 200 μg dose and analyzed *HTT*^Δ12^ mRNA levels from 7–112 days. Unexpectedly, the PK-PD relationship suggested increasing target engagement up to 56 days after dosing at a given tissue drug concentration (Figure 7D). The reasons for this outcome are unclear. It is possible that intracellular redistribution mechanisms, such as endosomal release and nuclear uptake, played roles in the result. However, further investigation is required to understand the exact mechanism involved.

Nevertheless, taken together, our results indicate that QRX-704 potently activates *HTT*^Δ12^ splicing in disease-relevant tissues after intracranial delivery. Although delayed maximum efficacy has to be taken into consideration, PK-PD follows a predictable relationship trend, and the results indicate a long tissue half-life, potentially supporting the need for infrequent dosing regimens.

### QRX-704 reduces the generation of N-terminal proteolytic fragments and the level of HTT aggregation with increase of the number of dendritic spines in the YAC128 HD mouse model.

**Figure 8:**
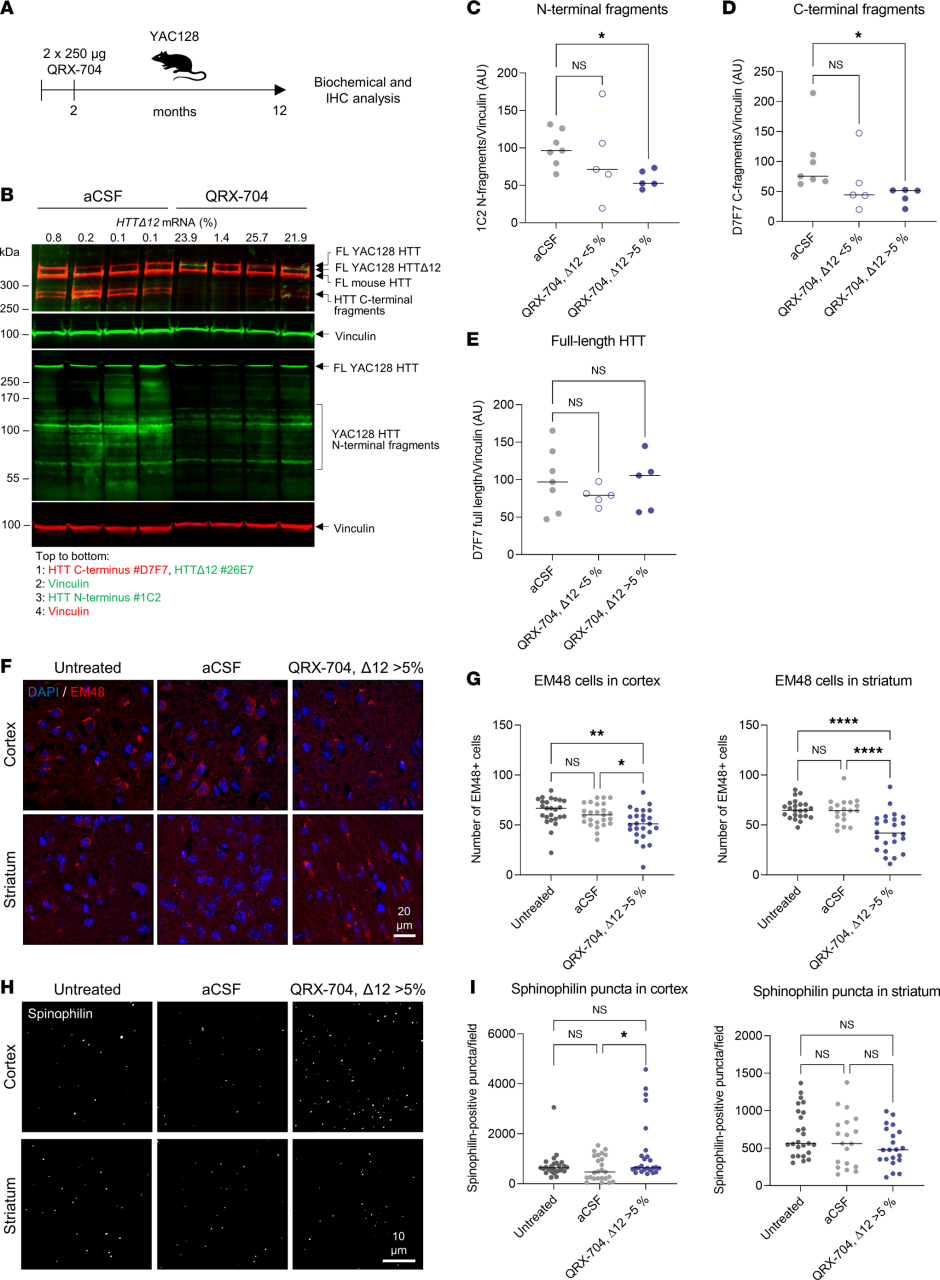
QRX-704 treatment of YAC128 mice reduces N-terminal cleavage fragments, alleviates HTT aggregation, and increases the number of dendritic spines. (**A**) Schematic of 10-month in vivo study performed in YAC128 mice dosed twice with 250 μg QRX-704 or aCSF (vehicle). (**B**) Western blot of different HTT species (HTT^Δ12^ or canonical isoform, full-length, C-terminal, and N-terminal fragments) of cortex from aCSF- or QRX-704–treated YAC128 mice with Abs as depicted in figure. *HTT^Δ12^* mRNA (% of total) in the same tissue sample for each animal is depicted above the top panel. (**C**) N-terminal fragments’ relative quantification (1C2 Ab) normalized by vinculin signal in animals treated with aCSF (gray, *n* = 7) or QRX-704 (blue), separated into animals with *HTT^Δ12^* mRNA less than 5% (open circles, *n* = 5) or greater than 5% (filled circles, *n* = 5), with mean ± SEM. (**D**) C-terminal fragments (D7F7 Ab) normalized by vinculin, analyzed as in **B**. (**E**) Full-length human HTT (D7F7 Ab) normalized by vinculin, analyzed as in **B**. (**F**) Immunostaining of sagittal brain sections of cortex and striatum from the right hemisphere of YAC128 mice from **A**. Representative images for HTT aggregation visualized by EM48 staining (red) and DAPI (blue) staining are shown for the indicated groups (*n* = 5 × 5 images recorded per location): untreated (dark gray), aCSF (light gray) and QRX-704 with HTT^Δ12^ greater than 5% (blue). (**G**) Quantification of the number of EM48-positive cells in recorded images from cortex and striatum. (**H**) The same as **F**, but immunostained for spinophilin indicating the number of dendritic spines. Representative images show spinophilin puncta, which were counted in an automated fashion. (**I**) Quantification of the number of spinophilin-positive puncta using ImageJ (NIH) threshold and counting tools on the recorded images from cortex and striatum. *P < 0.05, **P < 0.01, ****P < 0.0001. Statistics: 1-way ANOVA followed by Tukey’s multiple comparison test.

Most of the commonly used HD mouse models are based on either truncated N-terminal transgenes or exon 1 knock-in at the mouse *Htt* locus; in both cases, the critical splice site is absent. Hence, neither of these models allows the testing of QRX-704 activity. Therefore, we chose the FVB-YAC128 model to study the effect of QRX-704 treatment, despite the fact that it is characterized by a mild and slow phenotype with only moderately elevated caspase-6 activity compared with human HD (14, 16). We administered 2 repeated doses of 250 μg of QRX-704 or vehicle (aCSF) to 2-month-old FVB-YAC128 mice (Figure 8A); higher doses were not possible since the FVB-YAC128 line is prone to seizures. We analyzed *HTT* mRNA and HTT protein levels in cortical tissue 10 months after dosing. Some animals treated with QRX-704 displayed high levels of *HTT*^Δ12^ mRNA (Figure 8B), but we observed substantial heterogeneity within the group (0.4%–25.7%, median 5.0%). This high level of variability is in line with the PK-PD study results (Figure 7, B and C), in which large variability in QRX-704 tissue concentration (PK) per dose group was also observed, while the resulting PD effect (*HTT*^Δ12^ mRNA) was comparatively well predicted by the PK results. A plausible explanation for the variable tissue drug concentration is a variable success rate of dosing. Subsequently, to be able to separately evaluate the potential effects based on samples originating from mice with low or high *HTT exon12* skipping levels, we stratified the samples into groups with less than 5% or greater than 5% *HTT*^Δ12^ mRNA in a post hoc analysis. Notwithstanding the potential bias of this approach, we found that animals with greater than 5% *HTT*^Δ12^ displayed a decrease in N-terminal fragments, as detected with the 1C2 polyQ-specific Ab (Figure 8C). In line with this finding, the levels of C-terminal fragments of approximately 280 kDa were decreased (Figure 8D), while those of full-length HTT were unchanged (Figure 8E).

Since mHTT proteolysis is significantly reduced upon QRX-704 treatment, we tested the behavioral consequences in QRX-704–treated FVB-YAC128 mice. Although YAC-128 mice show only moderate behavioral alterations, 1 study reported a depression-like phenotype in the Porsolt swimming test, and this behavior that was reestablished when caspase-6 cleavage was inhibited in the so-called C6R line (14). We subjected the QRX-704–treated animals to the swimming test but did not observe significant differences between the YAC-128–treated animals and the vehicle-treated controls (Supplemental Figure 7).

Even though we could not detect significant changes in the behavioral tests performed, we analyzed brain tissue from the same mice by IHC. When brain sections were analyzed for HTT protein aggregation using EM48 Ab (40–42), we observed a significant decrease in HTT aggregation in QRX-704–treated mice with greater than 5% mRNA (Figure 8, F and G). We quantified cells showing positivity for EM48 staining in the cortex and striatum in brain sections from the mice described above (Figure 8A) and observed a lower number of EM48-positive cells in both the cortex and striatum (Figure 8G). In addition, we investigated the presence of spinophilin-positive puncta, a phosphatase 1-binding protein localized primarily in dendritic spines, which were previously shown to be reduced in the cortex of HD mice (43). Within the sections from the cortex from the group of QRX-704–treated mice with greater than 5% HTT^Δ12^, we detected a significant increase in spinophilin-positive puncta, indicating an increase in dendritic spines (Figure, 8, H and I).

Taken together, our findings indicate that the QRX-704–generated polyQ-expanded HTT^Δ12^ protein is protected from D586 cleavage in vivo and from subsequent downstream proteolytic events. We could detect a phenotypic improvement in the form of decreased HTT aggregation and an increased dendritic spine count, while we could not demonstrate beneficial effects on the behavior of YAC128 mice, which may require long-term observation. In addition, QRX-704 had no deleterious effect in these mutant HD mice over a treatment period of 10 months.

## Discussion

Here, we present a systematic analysis of the structural and functional equivalence of HTT^Δ12^ compared with normal HTT, which provides the cornerstone of the QRX-704 therapeutic approach for attenuating HD pathology.

Our in vitro caspase-6 cleavage assays showed that HTT^Δ12^ is resistant to caspase-6 cleavage. HTT^Δ12^ has a 45 aa deletion located at the N-terminus in the proximity of the caspase-6 cleavage site (D586; the numbering follows Q23HTT). The deletion converts the original caspase-6 site IVLD_586_G to QVLD_586_G. It has been shown that the P4 position of the caspase-6 cleavage site containing hydrophobic aa is preferred for efficient cleavage (44). HTT^Δ12^ has a hydrophilic aa (Q, glutamine) at the P4 position, making HTT^Δ12^ resistant to caspase-6 cleavage. The truncation of exon 12 in HTT is unlikely to affect the structure and function of HTT due to the size of the deletion (a 45 aa deletion within the full-length HTT sequence of over 3,144 aa) and the fact that exon 12, which contains the caspase-6 site, is located in the disordered region within the N-HEAT domain of HTT (27).

A crucial prerequisite of the use of QRX-704 strategy for treating patients with HD is the demonstration that this approach is safe. While the truncation of exon 12 generated by QRX-704 is likely to be protective as it leads to reduced pathogenic proteolysis, we deemed it essential to assess whether the exon 12 truncation can induce an alteration of the function of wtHTT. Indeed, recent clinical trials aiming to reduce mHTT levels had to be interrupted due to unfavorable benefit/risk profiles for the study patients (45). While long-term adverse effects of HTT-lowering drugs have been proposed, one of the explanations for the worsening of the patients treated with the ASO drug tominersen may be linked to the fact that the applied ASO does not discriminate between wt and mutant alleles, and thus, also affects the level of wtHTT, which has a neuroprotective function (1).

First, we showed that the HTT and HTT^Δ12^ proteins have overall similar biophysical properties. Our structural analysis, including CD and cryo-EM results, shows the overall similarity of the structures in HTT and HTT^Δ12^, indicating that the exon 12 truncation does not affect the structure of HTT.

Second, we generated a mouse model (*Htt*^Δ12^) with a genomic 135 nucleotide deletion, mimicking the exon 12 human splice isoform. While homozygous *Htt*-null mouse embryos die (29, 46, 47), animals homozygous for the *Htt*^Δ12^ allele develop normally and are born at the expected Mendelian ratio; moreover, they are fertile and survive for at least 16 months. Neither a transcriptome analysis nor cortical development assessment led to the identification of significant differences in these mice from their wt littermates. The results indicate that the HTT^Δ12^ isoform can functionally replace the canonical isoform and maintain critical HTT-associated functions during mouse embryonic development and in adult physiology.

Third, we extensively assessed the functions of HTT, whose alteration affects cellular functions in vitro and in vivo. These functions include the axonal transport of BDNF-containing vesicles, whose defects reduce neurotrophic support to the striatum (7). The findings also suggest that the truncation of exon 12 in HTT does not affect its interaction with molecular motors, i.e., dynein or kinesin (1), and thus support our findings that the exon 12 truncation does not profoundly modify the HTT structure or its conformation. This result was confirmed by the analysis of Golgi reassembly, which depends on the activity of the dynein motor (48) and the interaction between HTT and dynein (37, 38). We also observed a lack of an effect of exon 12 truncation on HTT function in ciliogenesis. These findings are important, given that a lack of wtHTT reduces ciliogenesis, alters CSF circulation, and leads to hydrocephaly in mice (9). While it remains possible that the exon 12 truncation is associated with a specific function that remains to be identified, our findings suggest that exon 12 truncation does not induce obvious deleterious effects on the known canonical functions of HTT. Taken together, the complete set of structural, biochemical and functional studies that we have performed of the HTT^Δ12^ isoform in vitro and in vivo support the notion that HTT^Δ12^ maintains normal HTT structure and function, indicating that, in this regard, the QRX-704 approach appears safe.

We found that QRX-704 is well tolerated and showed predictable PKs with a tissue half-life of 4–8 months, which is within in the same range reported for Nusinersen (Spinraza), a market-approved compound for the treatment of spinal muscular atrophy with oligonucleotide chemistry similar to that of QRX-704. In the FVB-YAC128 mouse model, QRX-704 induced splicing-switching to the *HTT*^Δ12^ transcript in a dose-dependent manner. Our study was limited by our capacity to assess phenotypic consequences in the YAC128 animals given their mild phenotype and by the variability of dosing that induced, consequently, a variability in the observed effects. When subgrouping the animals based on the median effect in the production of the HTT^Δ12^ transcript, we observed significant reductions (Figure 8, C, D, G and I) in the production of N- and C-terminal fragments and showed that QRX-704 induced improvements in HTT aggregation and dendritic spine counts. While this approach has clear limitations and was underpowered due to the unexpected variability, our results suggest that QRX-704 treatment has the potential to alleviate HD-associated pathology when sufficient dosing is achieved. It is plausible that higher doses or more dose repetitions (2 separate 250 μg doses tested here) would generate sufficiently high efficacy in all treated animals needed to observe phenotypic effects on the full treatment group level, as the current data set and post hoc analysis have a risk of bias.

Another critical problem we faced in assessing the therapeutic effects of QRX-704 on HD-related phenotypes is the lack of suitable animal models. While a plethora of both rodent and larger animal models exist, the majority are cDNA-based transgenic models that do not allow the testing of splice-modifying therapies. Other models, such as the phenotypically relevant Q175 model and its derivatives, are based on human exon 1 knock-in at the mouse *Htt* locus. Thus, these models do not harbor the required *HTT*^Δ12^ splice site in exon 12 that is selectively present in primates and bat species. The YAC128 and BACHD models, together with the so-called humanized models developed by the Hayden group, are the only existing models that meet the requirements for testing a drug such as QRX-704. We chose the YAC128 model because of its moderately more pronounced and well-documented phenotype. However, this model is imperfect in that the caspase-6 activity level is relatively low in these mice compared with the highly elevated activity levels of caspase-6 observed in patients with HD (16). A better suited HD model would mimic elevated caspase-6 activity to allow a more robust assessment of QRX-704. A detailed characterization of levels and identities of specific N-terminal and C-terminal HTT fragments in a relevant HD model where caspase-6 activity is elevated would be relevant to understand the full picture of the downstream proteolytic cascade. Another caveat of our work using the YAC128 model is that YAC128 is not well suited for testing whether QRX-704 treatment alleviates contributing pathology caused by HTT loss of function, since the transgene is overexpressed in a background of 2 copies of endogenous mouse *Htt*. Crossing the YAC128 transgene in an *Htt*-null background did exacerbate its phenotype (2), supporting the loss-of-function hypothesis. This model or the Hu128/18 (49) could be relevant models for this purpose.

In summary, we have demonstrated a pharmacologically feasible ASO-based therapeutic approach, generating an alternative HTT isoform, which is structurally, biochemically, and functionally intact in important neuroprotective HTT functions but is resistant to caspase-6 cleavage at the critical D586 position. Treatment of YAC128 mice with QRX-704 reduced the formation of toxic N-terminal fragments as predicted by the sequential cleavage hypothesis and subsequent HTT aggregates. This approach may be a therapeutic strategy for the treatment of HD that increases the amount of protective full-length HTT while preventing the formation of toxic N-terminal and C-terminal HTT fragments.

## Methods

Detailed methods can be found in Supplemental Methods. Data have been deposited in National Center for Biotechnology Information’s Gene Expression Omnibus and are accessible through GEO accession number GSE209893.

### Study approval.

Mouse experiments were approved by the Animal Welfare Body (Instantie voor Dierenwelzijn, Utrecht, the Netherlands), the Animal Tests Committee (Dierexperimentencommissie, Leiden, the Netherlands), and the Central Animal Testing Committee (Centrale Commissie Dierproeven, Den Haag, the Netherlands) and conformed to the European Community regulations (Directive 86/609/EEC).

## Author contributions

HK and YL performed biochemical and structural characterization of HTTs; and TJ and PP performed cryo-EM analysis. SL performed the in vitro studies of the HTT^Δ12^ functionality. AH, ZKK, and FVDH performed ex vivo analysis. WB, GBVDH, HA, and LCMB performed in vivo experiments. PK performed COS7 biochemistry and analyzed PK-PD. GJP oversaw the project within ProQR. All authors reviewed the data. HH, FS, SH, PK, and JJS led the project. HK, SL, AH, HH, FS, SH, PK, and JJS wrote the manuscript.

## Supplementary Material

Supplemental data

## Figures and Tables

**Table 1:**
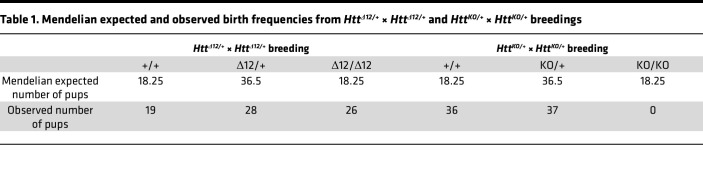
Mendelian expected and observed birth frequencies from *Htt^Δ12/+^* × *Htt^Δ12/+^* and *Htt^KO/+^* × *Htt^KO/+^* breedings

## Version 1 08/09/2022

In-Press Preview

## Version 2 09/08/2022

Electronic publication
