# Supplementary material for: A pathogenic proteolysis–resistant huntingtin isoform induced by an antisense oligonucleotide maintains huntingtin function
Source: JCI Insight. 2022 Sep 8;7(17):e154108. doi: 10.1172/jci.insight.154108 (PMC9536263; doi:10.1172/jci.insight.154108)
Supplement: Supplemental data [file jciinsight-7-154108-s081.pdf]

Supplemenatary Information

Figure S1

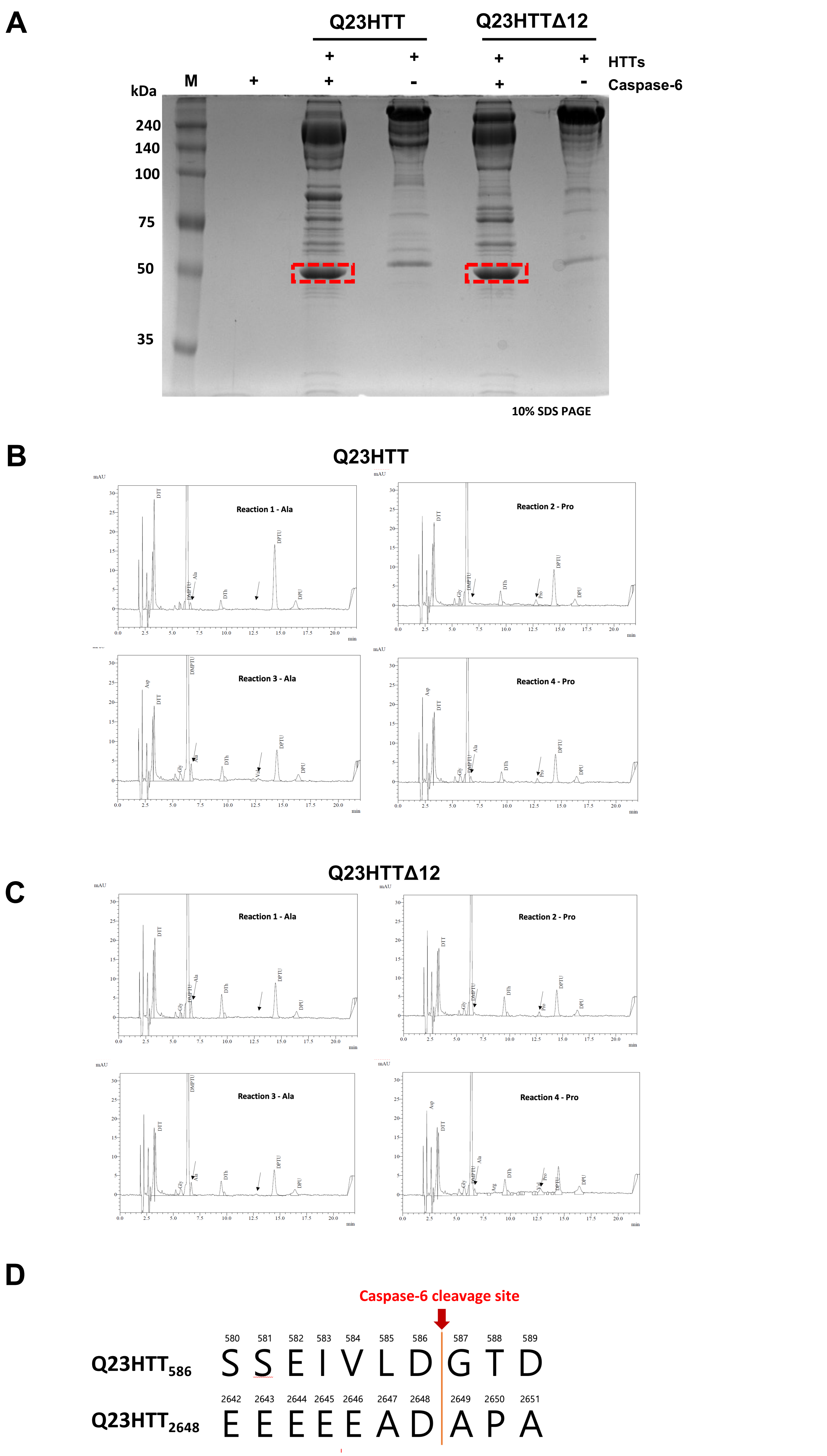
A
Q23HTT
Q23HTTΔ12
HTTs
Caspase-6
kDa
M
240
140
100
75
50
35
10% SDS PAGE
B
Q23HTT
Reaction 1 - Ala
Reaction 2 - Pro
Reaction 3 - Ala
Reaction 4 - Pro
C
Q23HTTΔ12
Reaction 1 - Ala
Reaction 2 - Pro
Reaction 3 - Ala
Reaction 4 - Pro
D
Caspase-6 cleavage site
Q23HTT586
580 581 582 583 584 585 586 587 588 589
S S E I V L D G T D
Q23HTT2648
2642 2643 2644 2645 2646 2647 2648 2649 2650 2651
E E E E E A D A P A

Figure S1. Identification of the second caspase-6 cleavage site at C-terminal region in HTT

(A) Caspase-6 treatment generates a fragment corresponding to 50 kDa. The bands indicated with red dotted box were subjected to N-terminal sequencing. **(B, C)** HPLC Chromatographs for Q23HTT (B) and Q23HTTΔ12 (C) from N-terminal sequencing. **(D)** Caspase-6 cleavage sites at N-terminal ($EIVLD_{586}$) and C-terminal region ($EEEAD_{2648}$).

Figure S2

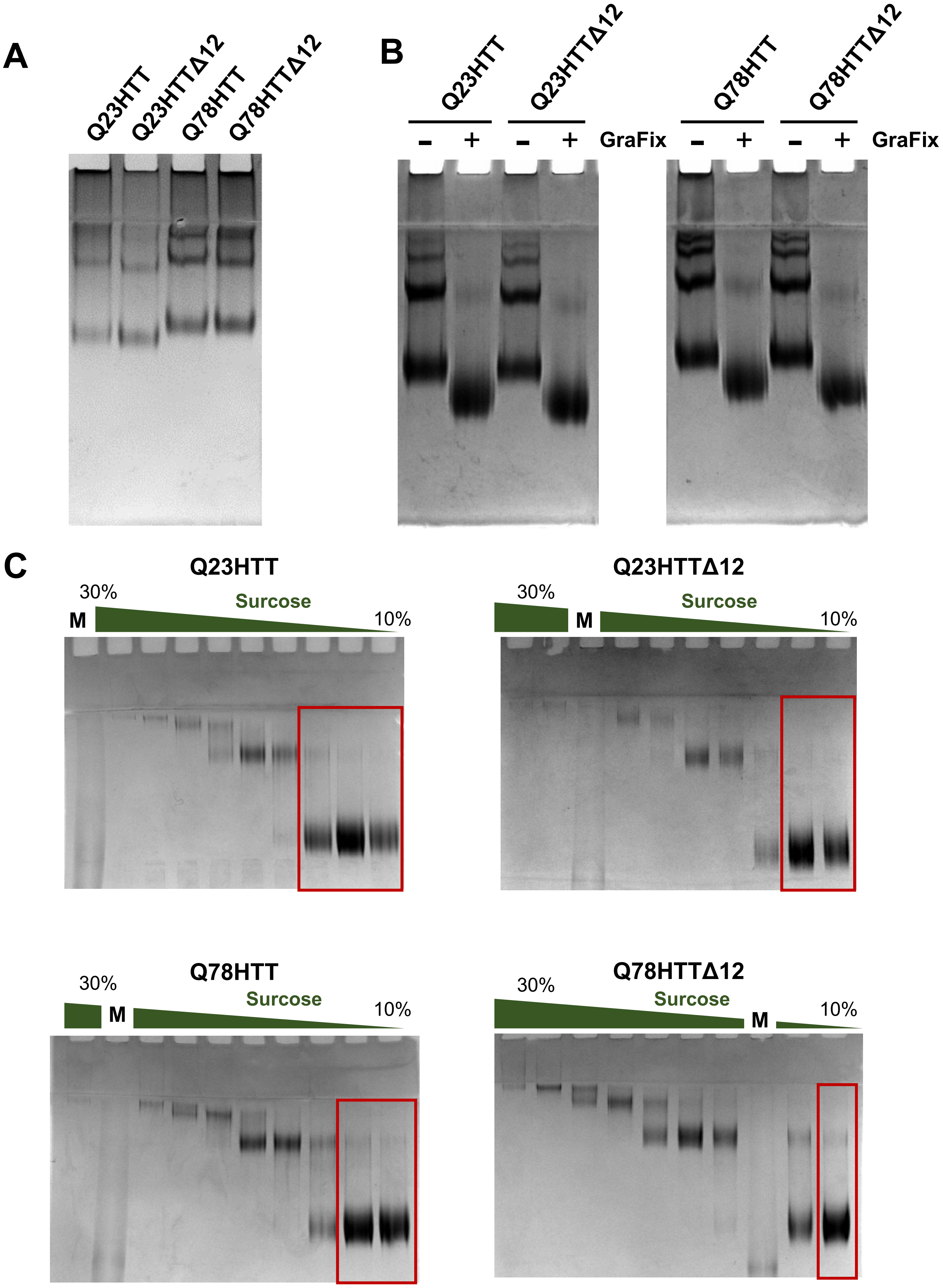

Figure S2. Purification of huntingtins

(A) 5% Native PAGE analysis of purified huntingtins (Q23HTT, Q23HTTΔ12, Q78HTT, Q78HTTΔ12). **(B)** 5% Native PAGE analysis of monomeric huntingtins separated by GraFix (+). **(C)** Sucrose gradient (30-10%) ultracentrifugation of HTTs. The fractions were analyzed in 5% native-PAGE. The red boxes were pooled for monomeric HTTs.

Figure S3

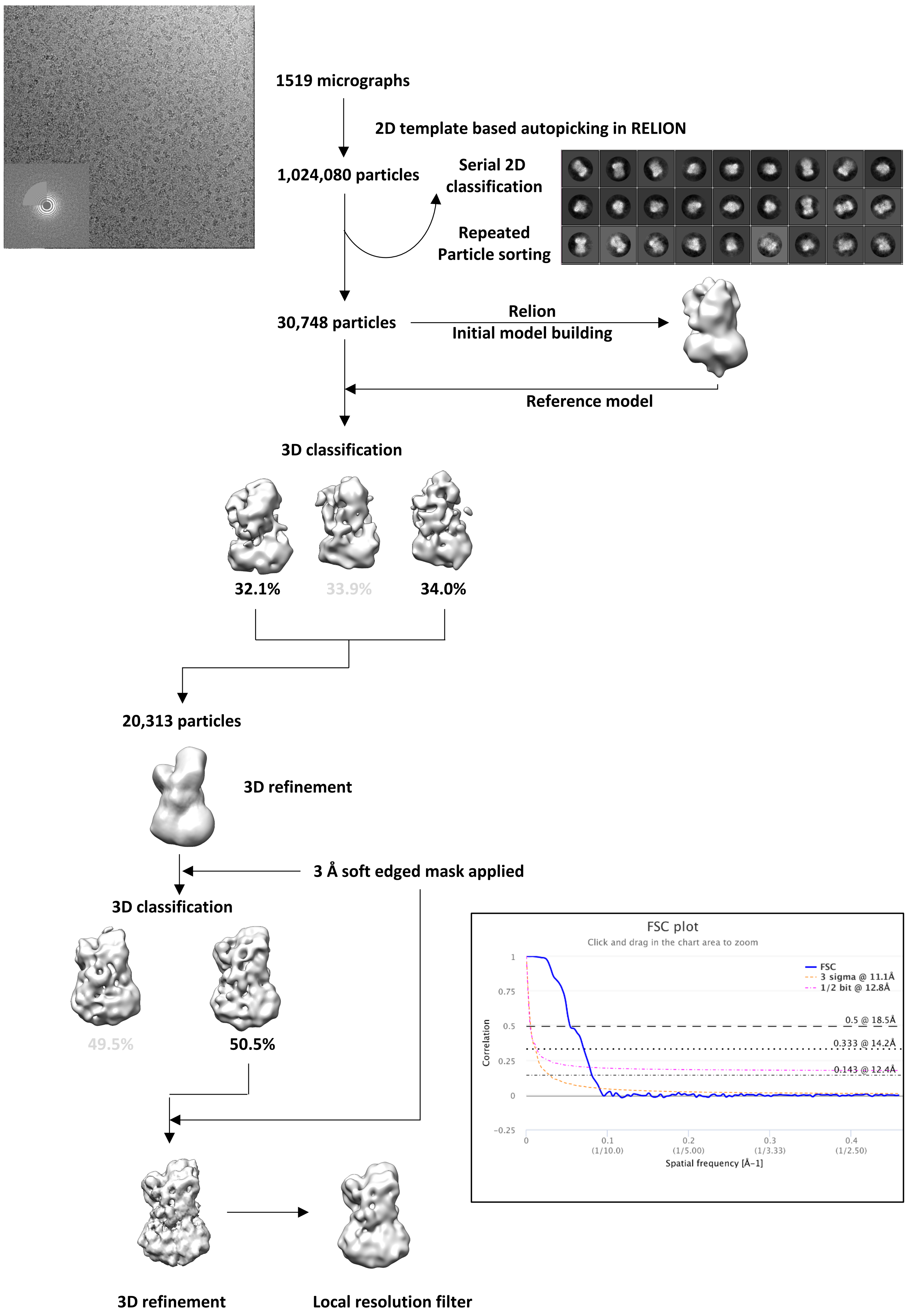

Figure S3. Cryo-EM image processing of Q23HTTΔ12
A scheme of cryo-EM image processing for Q23HTTΔ12 and the corresponding Fourier shell correlation plot.

Figure S4

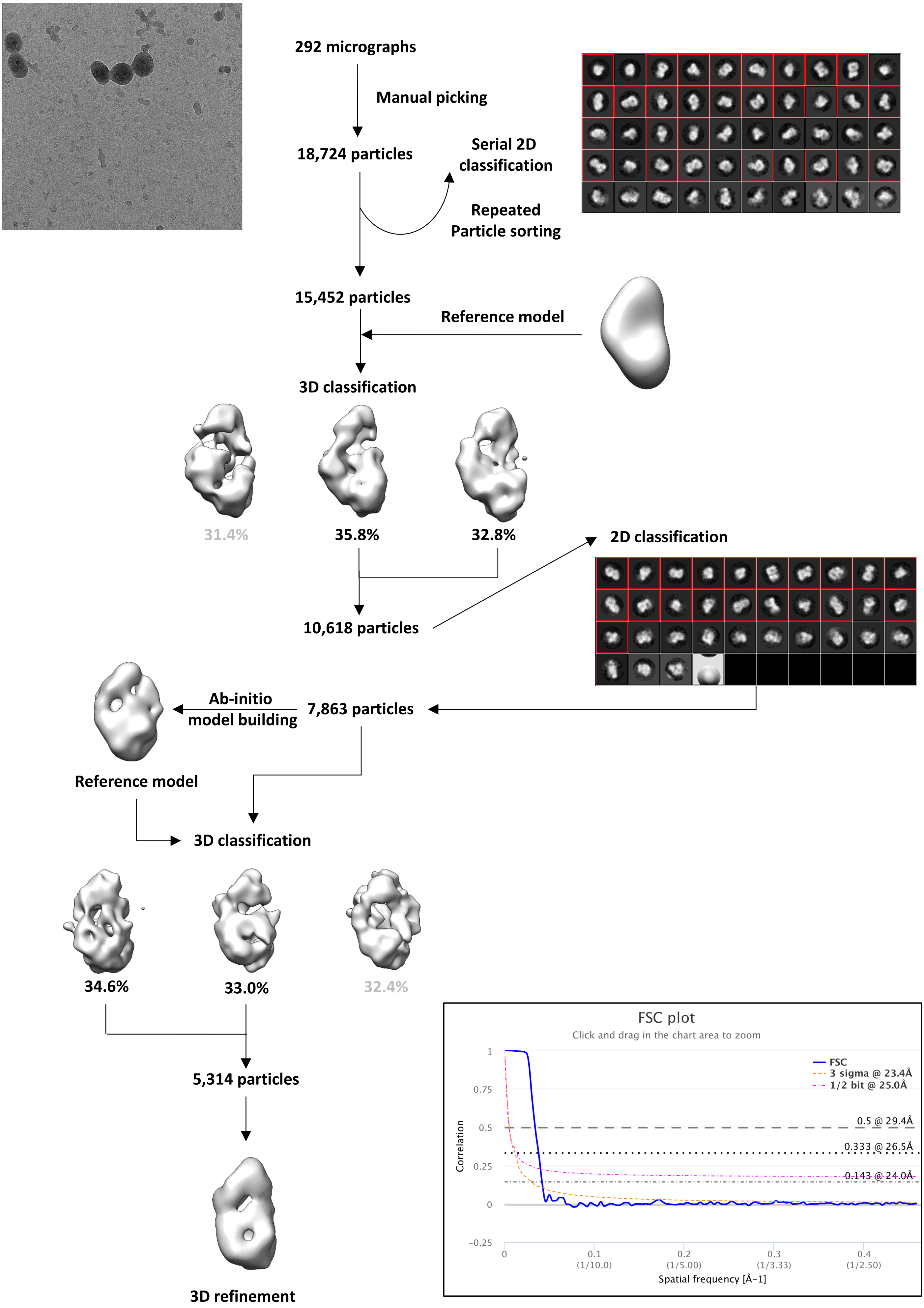

Figure S4. Cryo-EM image processing of Q78HTTΔ12
A scheme of cryo-EM image processing for Q78HTTΔ12 and the corresponding Fourier shell correlation plot.

Figure S5

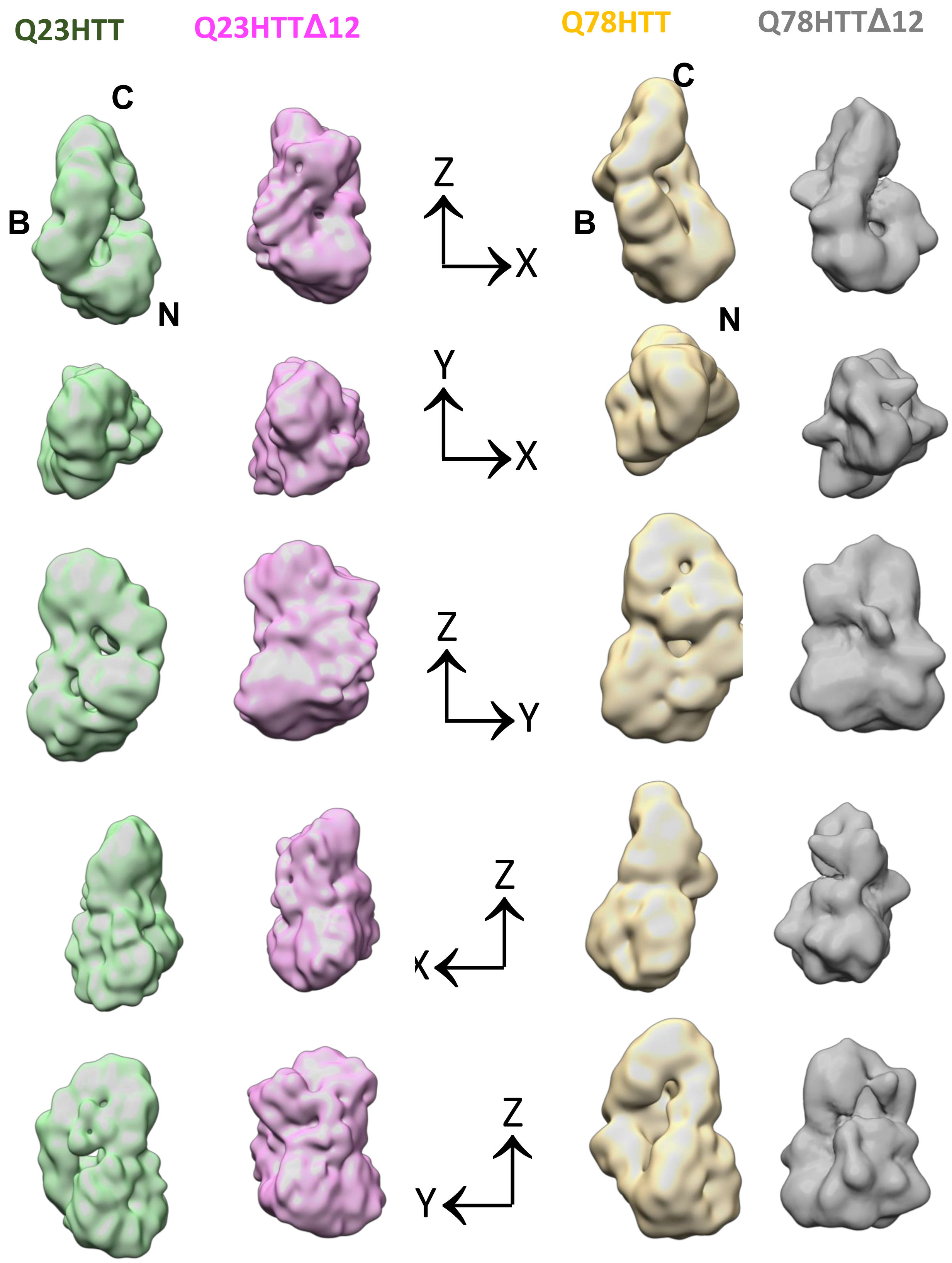

Figure S5. Structural comparison of HTTs and HTTΔ12s
3D cryo-EM maps of Q23HTTΔ12 (pink) and Q78HTTΔ12 (gray) are compared to the corresponding wild type structures (Q23HTT in green and Q78HTT in yellow) at five different orientations (26). N, B and C depict approximate locations of the N-, Bridge and C-domains.

Figure S6

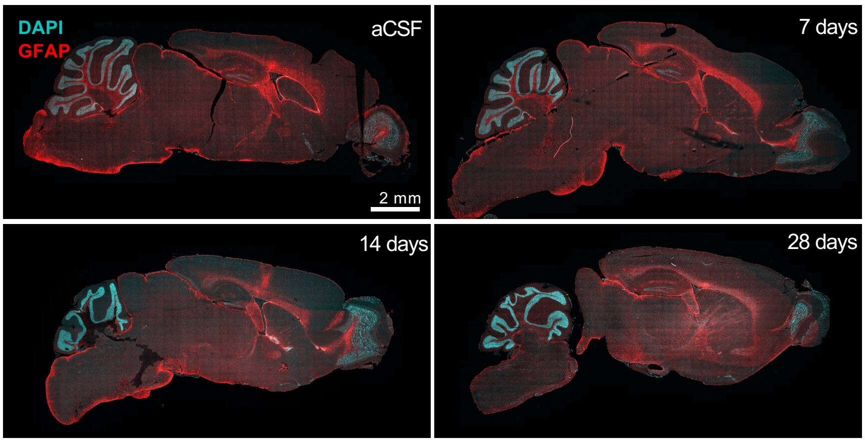

Figure S6. GFAP immunofluorescence in wildtype mice treated with QRX-704

Representative images showing GFAP (*Glial Fibrillary Acidic Protein*) (red) and DAPI (blue) immunofluorescence staining of brain sections derived from brain tissue of QRX-704 treated or untreated (aCSF) YAC128 mice at indicated timepoints. Scale bar 2 mm.

Figure S7

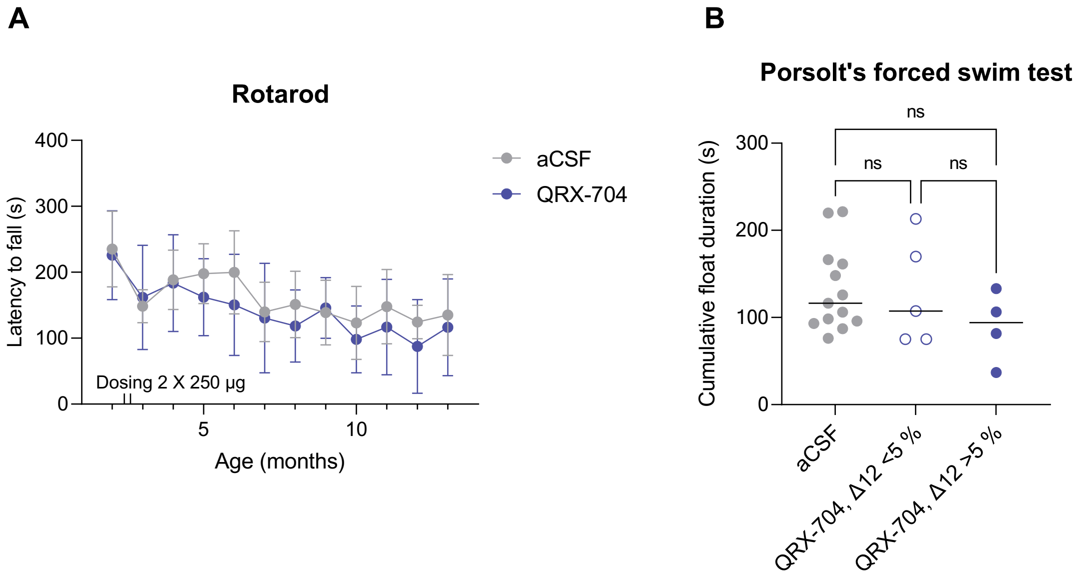

Figure S7. Behaviour assessment in YAC128 mice treated with QRX-704 or aCSF
(A) Monthly rodarod tests of motor coordination from 2 to 13 months age of YAC128 mice treated with 2 x 250 µg QRX-704 or aCSF (vehicle) dosed at 10 weeks age, depicting latency to fall (seconds), lower indicative of YAC128 HD associated phenotype (median +/- standard deviation). **(B)** Porsolt's forced swim test of animals of the study in (A) performed at 13 months age, depicting cumulative float duration in seconds, higher indicative of YAC128 HD associated phenotype. QRX-704 treated animals are presented as animals with cortical Δ12 mRNA>5 % or <5 % (analyzed after sacrificing). ns = non-significant, Kruskal-Wallis with Dunn's multiple comparisons test. N=9-12 animals per group.

Table S1. Cryo-EM data collection and image processing statistics

	**Q23HTTΔ12**	**Q78HTTΔ12**
**Sample Preparation**		
Grid type	Quantifoil 2/2	Quantifoil 2/2
Cryo-specimen freezing	Vitrobot 2	Vitrobot 2
**Data Collection**		
Microscope	FEI Titan KRIOS G3i	
Detector	Gatan K2 summit (Electron Counting Mode)	
Total Electron Exposure ($e^-/Å^2$)	44.8	44.8
Dose rate ($e^-/Å^2$/sec)	5.6	5.6
Number of fractions	40	40
Exposure time (sec)	8	8
Defocus range (μm)	-1.4 ~ -3.5	-1.4 ~ -3.4
Pixel size (Å)	1.09	1.09
**Image processing**		
Software	RELION2.1	RELION2.1
Initial number of particles	1,024,080	18,724
Final number of particles	10,036	5,314
Symmetry imposed	C1	C1
Map resolution (Å) (Gold Standard FSC 0.143)	12.4	24

Table S2. A list of primers and probes

Primer name	Sequence
Fw for wt HTT	5'-GAC-AGC-TCC-CAG-ACC-ACC-ACC-3'
Rv primer for wt HTT	5'-AGG-CTG-CCT-GCA-GTG-ACT-CAT-3'
Fw primer for Δ12 HTT	5'-GCC-ACT-GAT-GGG-GAT-GAG-GAG-GA-3'
Rv primer for Δ12 HTT	5'-ATG-TGC-CTG-TTG-AAG-GGC-CAT-GG-3'
Fw primer for total HTT	5'-ACC-AGT-ATT-TGG-GCC-TGC-AGA-3'
Rv primer for total HTT	5'-AGG-CTG-CCT-GCA-GTG-ACT-CAT-3'
Fw primer mHPRT1	5'-TCA GTC AAC GGG GGA CAT AAA-3'
Rv primer mHPRT1	5'-GGG GCT GTA CTG CTT AAC CAG-3'

Probe name	Mod5'	Sequence	Mod3'
wt HTT	5'-/5HEX/	CCC-CTT-CAG-ACA-GTT-CTG-AAA-TTG-TGT-T	/3BHQ_1/-3'
HTTΔ12	5'-/5HEX/	CCA-GCC-AGG/ZEN/TGT-TAG-ACG-G	/3IABkFQ-3'
Total HTT	5'-/5HEX/	AGG-AAC-TCT-TCC-ATG-GCC-CTT-C	/3BHQ_1/-3'
mHPRT1	5'-/56-FAM/	GGT-GGA-GAT/ZEN/GAT-CTC-TCA-ACT-TTA-ACT	/3IABkFQ-3'

Supplemental Data 1

Statistical analyses

Figure 4:

**Fig4 C-E** Kymograph quantification	Segmental velocity (µm/s) ± SEM		Mean number of vesicles (/100 µm/30s) ± SEM			Number of axons analyzed
	anterograde	retrograde	anterograde	retrograde	pausing	
HTT	2.341 ± 0.07816	-1.781 ± 0.05147	16.30 ± 0.9521	-12.33 ± 0.8516	9.649 ± 0.5492	84 (from 5 different embryos in 2 independent cultures)
HTTΔ12	2.291 ± 0.06908	-1.868 ± 0.05126	16.56 ± 1.073	-13.27 ± 0.93	10.41 ± 0.6966	84 (from 5 different embryos in 2 independent cultures)
Mann-Whitney test: HTT vs HTTΔ12	ns p= 0.8303	ns p=0.2076	ns p=0.9106	ns p=0.6635	ns p=0.6890	

**Fig4 F-G** Kymograph quantification	Mean linear flow (µm/s) ± SEM	Mean directional flux (µm/s) ± SEM	Number of axons analyzed
HTT	64.82 ± 4.564	16.46 ± 2.719	84 (from 5 different embryos in 2 independent cultures)
HTTΔ12	66.11 ± 4.918	13.67 ± 2.01	84 (from 5 different embryos in 2 independent cultures)
Mann-Whitney test: HTT vs HTTΔ12	ns p=0.8780	ns p=0.6498	

Figure 5:

**Fig5 D**	Average size of Golgi particles (µm²) ± SEM			
	T0	T60	T90	T120
Scrb	0.7824<br>± 0.1086	7.361<br>± 0.1473	8.339<br>± 0.1303	9.814<br>± 1.174
ASO	0.6939<br>± 0.05502	5.986<br>± 0.5	6.929<br>± 0.3242	8.194<br>± 0.677
Number of cultures	7 Scrb<br>9 ASO	8 Scrb<br>11 ASO	8 Scrb<br>8 ASO	4 Scrb<br>8 ASO
T-test: Scbr vs ASO	ns p=0.4492	* p=0.0361	** p=0.0012	ns p=0.2269

**Fig5 F**	Average size of Golgi particles (µm²) ± SEM			
	T0	T60	T90	T120
HTT	0.6153<br>±0.05595	8.37<br>±1.395	8.953<br>±1.203	8.656<br>±0.7359
HTTΔ12	0.5916<br>±0.04848	7.267<br>±0.7369	7.883<br>±0.7324	9.337<br>±0.7547
Number of cultures	8 HTT<br>9 HTTΔ12	7 HTT<br>9 HTTΔ12	7 HTT<br>9 HTTΔ12	7 HTT<br>7 HTTΔ12
Mann-Whitney test: HTT vs HTTΔ12	ns p=0.9085	ns p=0.8371	ns p=0.5360	ns p=0.5350

Figure 6:

**Fig6 B**	Percentage of cells with cilia (%) ± SEM
Scrb	70.06±2.632
ASO	60.18±2.802
Number of ROI from 7 cultures from 4 independent experiments	59 Scrb<br>59 ASO
T-test: Scbr vs ASO	*p=0.0114

**Fig6 D**	Percentage of cells with cilia (%) ± SEM
HTT	72.40±2.948
HTTΔ12	66.50±2.699
Number of ROI from 6 to 8 different cultures from 3 independent experiments	46 HTT<br>62HTTΔ12
T-test: HTT vs HTTΔ12	ns p=0.1463

Supplemental Methods

Purifications of recombinant full-length HTTs

Recombinant full-length HTTs (Q23HTT, Q78HTT, Q23HTTΔ12 and Q78HTTΔ12) were all purified using our estiablished protocol (28, 49). In short, HTTs with N-terminal FLAG tag was expressed in Sf9 insect cells (ThermoFisher Scientitifc, 11496015) infected with Baculovirus. HTTs were purified with M2 resin (Sigma-Aldrich) followed by Superdex 200 26/60 size-exclusion column.

Purifications of recombinant caspase-6

Caspase-6 (residues 24-293) (without the prodomain) was cloned into the NheI/XhoI sites of pET21a vector with C-terminal His-tag. Caspase-6 was expressed in BL21(DE3) E.coli for expression by adding 0.5mM Isopropyl β-D-1-thiogalactopyranoside (IPTG) at 1 OD_{600} for 18 hrs at 24℃. The cells were then collected by centrifugation at 4,600xg for 20 min and the cell pellets were resuspended with 30 ml of a buffer (Buffer A) containing 100mM NaCl, 50mM Tris-HCl (pH8.0) and 5% Glycerol. The resuspended cells were broken with sonication and the cell debris were clarified by centrifugation at 27,000xg for 1hr. The supernatants were first purified with Ni-NTA affinity chromatography resin and the resin was washed with the buffer A additionally containing 70 mM Imidazole. The proteins were eluted from the resins with a buffer containing 100 mM NaCl, 50 mM Tris-HCl (pH8.0) and 300mM Imidazole. Just before size exclusion chromatography, the pH of the eluant containing the protein was adjusted to 5.0 with acetic acid. The protein was further purified with a Superdex® 75 Increase 10/300 column (GE Healthcare) equilibrated with a buffer containing 50 mM NaCl, 20 mM Sodium acetate (pH 5.5) and 10mM dithiothreitol (DTT). The proteins were concentrated with an Amicon Ultra 10kDa filter to 0.4 mg/ml. The final samples were stored at -20℃ in the presence of 50% Glycerol.

In vitro caspase-6 cleavage assay

In vitro caspase-6 cleavage assays were performed in a reaction (20 µl) containing 8 μg recombinant HTTs and various amounts of recombinant caspase-6 (12.5 ng, 25 ng, 50 ng or 100 ng) in the presence of 50 mM NaCl, 20 mM HEPES-HCl (pH7.5), 2 mM EDTA, 0.1 % CHAPS and 5 mM DTT. The reactions were then incubated at 37℃ for 30 min and stopped with a SDS loading buffer. The cleaved products were visualized with 10% SDS-PAGE gel stained by coomassie-blue staining.

N-terminal sequencing

For identifying the N-terminal end of the caspase-6 cleaved fragments, 20 μg of recombinant HTTs were incubated with 2 μg of recombinant caspase-6 following the same protocol as described above. Cleaved products were separated with 10% SDS-PAGE and transferred to a PVDF membrane. The protein bands on the PVDF membrane were visualized with coomassie-blue staining. The target fragment band was cut and loaded onto an automated N-terminal amino acid sequencing performed with PPSQ-51A (SHIMADZU) equipped with an HPLC system. All reagents and solvents used in the equipment were purchased from WAKO chemical.

COS7 caspase-6 assay

COS7 cells (ATCC, CRL-1651) were transfected with HTT wt or HTTΔ12 plasmids using Lipofectamine 2000 (ThermoFisher Scientific) for 24 hrs. Cells were collected in caspase assay buffer containing 50 mM HEPES, 50 mM NaCl, 10 mM EDTA and 0.1% 3-[(3-cholamidopropyl) dimethylammonio]-1-propanesulfonate (CHAPS). Protein concentration was determined using the BCA assay as above, 10 mM DTT was added to the lysates, and reactions containing 5 µg total protein were setup with 0-4.0 units Caspase-6 (Biovision Cat no 1086-100), incubated at 37C for 5 hrs.

Circular Dichroism

CD spectra for purified recombinant HTTs at 1 mg/ml concentration were measured from 190-260 nm with a wavelength pitch 0.2 nm and a speed of 20nm/min. The samples were measured three times each to obtain the average spectra. For monomeric HTTs, HTTs were crosslinked with DSS and separated with ultra-centrifugation as described in our previous paper (28). CD spectra for the monomeric HTTs were measured in the same manner as described above.

CryoEM analysis

The separated Q23HTTΔ12 monomer preparations (3.5 µl of 0.08 mg/ml) were mixed with a final 0.05% concentration of octyl glucoside and loaded onto Quantifoil 2/2 200 mesh grids (Quantifoil) covered with graphene oxide (50). The grids were incubated with samples for 30 seconds, then vitrified by plunge freezing in liquid ethane using a Vitrobot II (Thermo Fisher Scientific) after 8~10 seconds blotting time. The vitrification chamber was kept at 15°C and 100% humidity respectively. Following transfer to liquid nitrogen, the grids were loaded into an autoloader of a Titan Krios microscope (Thermo Fisher Scientific) with a K2 Summit direct electron detector (Gatan) at the SciLifeLab Cryo-EM node, Umeå University. Purified Q78HTTΔ12 monomers were prepared in a similar way as Q23HTTΔ12. Details of the

imaging conditions and parameters are summarized in Supplementary Table S1. The collected data was imported into the Scipion platform for image processing (51). Initially the acquired movie frames were subjected to motion correction using MotionCor2 (52) and dose weighted and un-dose weighted micrographs were generated. The CTF estimation was done by using CTFFIND4 (53). Good images were selected based on estimated CTF resolution (< 8 Å), defocus range (1.4-3.5 μm), and the thickness of graphene oxide. All micrographs were thoroughly inspected and the particle picking was carried out using Autopick with a 2D template (Q23HTTΔ12) or manual picking (Q78HTTΔ12). The particles were extracted from dose weighted micrographs with a 250 x 250 pixel box size and the contrast was inverted for further image processing. The initial particle set was reduced by excluding outliers identified through several rounds of reference free Relion 2D classification (54) (Figures S3 and S4). The particles ending up in good 2D classes formed the basis for 3D initial model building using Relion 3D initial model (54). The initial maps (~ 20 Å resolution) were refined by repeated 3D classification and 3D autorefinement using Relion 2.0 (54) (Figure S3 and S4). UCSF-Chimera (55) was mainly used for further image analysis and validation. The estimated resolution was calculated from the EMDB FSC validation server (https://www.ebi.ac.uk/pdbe/emdb/validation/fsc/) using pairs of unfiltered half maps (Figure S3 and S4 and Table S1).

HTTΔ12 Plasmid Cloning

HTTΔ12 Plasmid was generated using gBlock® Gene Fragment (Integrated DNA Technologies®) according to the manufacturer's directions. A Δ135 gBlock® was subcloned into the Acc65I and BoxI restriction enzyme sites of vector pEZ-M02 (cat.nr. EX-Z2776-M02, GeneCopoeia™) HTT ORF. Generation of the HTTΔ12 Plasmid was confirmed by DNA sequencing.

Generation of *Htt*$^{\Delta 12}$ transgenic mouse lines

Transgenic lines were generated for ProQR by Charles River Genetically Engineered Animal Models & Services (GEMS), France using a CRISPR/Cas9 strategy. Briefly single guide RNAs (sgRNA) with proprietary sequences were designed and validated, targeting protospacer adjacent motif (PAM) sites in exon 12 and intron 12. A 123 nt single stranded donor DNA (ssDNA) oligonucleotide was designed, bridging the 135 bp deletion. sgRNAs, ssDNA and Cas9 protein were microinjected in C57BL6/N one-cell pre-implantation embryos and reimplanted into pseudo pregnant females. Founders were characterized by PCR and sanger sequencing and mated with wt animals. F1 animals carrying either the precise 135 bp deletion as a result of homologous-directed repair (*Htt*$^{\Delta 12}$), or a 147 bp deletion as result of non-homologous end joining, resulting in a frameshift (*Htt*^{KO}) were selected for further

experiments. Splicing and knockout phenotypes of the alleles were verified by RT-PCR and western blot. All subsequent breeding and tissue collection was performed by Charles River France.

HTTΔ12 26E7 antibody generation

The HTTΔ12 monoclonal antibody (26E7) was produced (Genscript®) by immunizing 10 mice with a synthetic peptide HSSSQVLDGT corresponding to the HTTΔ12 protein. After in-house testing of hybridoma clones and subclones, clone 26E7 was chosen to be further purified by protein A and peptide affinity chromatography (Gensrcript®).

Knock-in mouse brain western blot

Proteins were extracted with T-PER buffer (ThermoFisher Scientific, 87510) from whole Bl/6 mouse brains by homogenization with the Dounce tissue grinder set (Sigma-Aldrich, D8938). Protein concentration were measured by standard BCA assay (Pierce®) according to manufacturer's instructions. 3-8% Criterion™ TGX Protein gels from BioRad were used to load 40 µg of protein in NuPAGE LDS sample buffer (ThermoFisher Scientific) after it was denatured by heating to 95°C. Tricine (BioRad) was used as a running buffer and gels were run at a constant 100 V for ~ 5hrs. Proteins were transferred to an Immobilon-nitrocellulose 0.2 µm membrane and probed with the following antibodies: primary Huntingtin (D7F7) XP® Rabbit mAb (Cell Signaling Technology®, #5656, 1:2000); primary HTTΔ12 (26E7) Mouse mAb (in-house, 1:1000); secondary 800 CW goat anti-mouse (LI-COR Biosciences®, 1:10000); secondary 680 IRDye goat anti-rabbit (LI-COR Biosciences®, 1:10000).

Cryo-section preparation

Mouse brains from HTT KI mice and KO mice were extracted on day 7 after birth by Charles River. Brains were fixed and shipped in a 4% formalin solution at 4°C and subsequently transferred to 30% sucrose for 2 days. Prior to cryo-sectioning, brain halves were separated sagittally and frozen in Tissue-Tek O.C.T.™ compound (# 4583, Batch no. 0915500003, Exp date 2011-07). 10 µm sections were prepared using the Thermo Scientific cryotome FSE. Sections were dried at 37°C for 30 min and stored at 4°C until further handling.

Immunohistochemistry staining

IHC staining was performed following standard procedures. Shortly, sections underwent an antigen retrieval step when being incubated for 10 min at 95°C in a 0.01 M sodium citrate solution, pH 6, submerged in demi water and shortly dried at 37°C. Sections were permeabilized in 0.3% PBS-Triton X-100 for 30 min and blocked in 10% Normal Goat serum for 2 hrs. Primary antibody incubation was performed in 3% Normal Goat Serum in 0.15%

PBS-Triton X-100 buffer over night at 4°C using the following antibodies: mouse anti-Satb2 (ab51502) [1:100], rat anti-Ctip2 (Abcam, ab18465) [1:200] and rabbit anti-Tbr1 (Abcam, ab183032) [1:200]. 3 washing steps and secondary antibody incubation for 1 hr at RT followed in the same 3% Normal Goat Serum in 0.15% PBS-Triton X-100 buffer using Alexa Fluor^{TM} 488 goat anti mouse (Sigma-Aldrich, A11001, Lot# 1664729), 555 goat anti rat (Sigma-Aldrich, A21434, Lot# 1907302) and 568 goat anti rabbit (Sigma-Adrich, A11011, Lot# 1942295) secondary antibodies together with Hoechst 33342 (ThermoFisher Scientitifc , H3570, Lot# 1524924) [1:1000]. Sections were washed several times with PBS and subsequently mounted using Aqua Poly/Mount (Polysciences, 18606).

Confocal Imaging

Imaging was performed on a Zeiss LSM 800 confocal microscope using a Plan-Apochromat 20x/0.8 M27 objective and tile scan modus to generate overview images of the cortical region.

Image analysis cortical lamination measurement

ZEN software, ImageJ and CellProfiler were used for the confocal image analysis. An arbitrary measure using the line tool of the ZEN software was used to determine the length of different cortical layers defined by different cortical markers together with the total length of the cortical layer in µm. The average from the relative length of the left and right hemisphere was calculated and presented in a graph. Furthermore, equally sized section images were generated for the left and right hemisphere from the original ZEN file in ImageJ. A script for CellProfiler was written to exclude the background staining of the images, generating a nuclei mask for each image, and setting a threshold for each channel. The number of nuclei and the number of Satb2-/Ctip2-/Tpx2-expressing cells was calculated. The average of the left and right hemisphere was determined for measures and the5veragee amount of nuclei per analyzed section was presented in a graph. The same procedure was used to define the relative cortical layer thickness.

Transcriptomics analysis

Htt$^{\Delta 12/\Delta 12}$, *Htt*$^{\Delta 12/+}$, *Htt*$^{KO/+}$ and wt littermates from both strains, were generated from wt heterozygous breedings by Charles River France. 3 months animals were sacrificed, striata and cortices dissected and snap frozen. RNA extraction was performed as described below. Transcriptomics analysis was performed by RNA sequencing (GenomScan, Leiden, The Netherlands) using an Illumina NovaSeq 6000, with approximately 20 million paired reads per same, aligned to the Mus_musculus.GRCm38.p4.dna.primary_assembly reference using a short-read aligner based on Burrows—Wheeler Transform (Tophat v2.0.14) with

default settings. Data was analyzed using the DESeq2 package v1.14.1 within the R platform v3.3.0. to generate heatmaps of relative distances, and pairwise analysis of differentially expressed genes expressed as log2 fold change with Benjamini-Hochberg adjusted p-values.

RNA extraction, cDNA synthesis and ddPCR

Total RNA for RNAsequencing and droplet digital PCR (ddPCR) assays was extracted using the Rneasy Plus Mini Kit (QIAGEN®, ref#: 74136) according to the manufacturer's instructions. cDNA was synthesized using the ThermoFisher Scientific Maxima Reverse Transcriptase (ThermoFisher Scientific, ref#: EP0743 kit according to the Manufacturer's instructions, with Primer Oligo (dT) (ThermoFisher Scientific, ref#:SO131), and 500 ng RNA input. The Bio-Rad's QX200 system (Bio-Rad Laboratory, Hercules, California, USA) was used to perform droplet digital PCR (ddPCR). In each ddPCR reaction, two probes were used: a FAM-labeled probe for the reference mHPRT1 amplicon and a HEX-labeled probe for the target HTT (wt HTT; HTTΔ23; total HTT) amplicon. Total of three assays per sample, samples in every assay were analyzed in triplicate. Primers/probes used are listed in Table S2. The reaction volume was 20 Ml (10 µl 2× ddPCR Supermix for Probes [no dUTP], 0.5 µl 10× primers, 0.8 µl 25× probes, 2.4 µl nuclease-free water with 4 µl of cDNA). The primer and probe concentration was 100 µmol. PCR was performed in a T100 Thermal Cycler (BioRad) using the following conditions: 95°C for 10 min, followed by 40 cycles of 95°C for 30 s, and 57.5°C for 60 s with a final stage at 98°C for 10 min. After thermal cycling, the 96-well plate was read in the QX200 Droplet Reader, and based on positive droplets and according to the Poisson distribution, the absolute copy number of the target amplicon was calculated using the QuantaSoft analysis software (Bio-Rad Laboratory, Hercules, California, USA). The data have been deposited in NCBI's Gene Expression Omnibus and are accessible through GEO accession number GSE209893.

Primary neuronal culture in microfluidic devices

Microfluidic devices were generated as previously described (56). Briefly, cortical primary cultures were performed from E15.5 *Htt*$^{\Delta 12/\Delta 12}$ knock-in and wt embryos. Dissociated cortical neurons were re-suspended in growing medium and plated in the chamber with a final density of ~7000 cells/mm^2 using growing medium (Neurobasal medium supplemented with 2% B27, 2 mM Glutamax, and 1% penicillin/streptomycin (ThermoFisher Scientific, 21103049, 17504044, 35050038 and 15140122) and then placed at 37°C and 5% CO_2. At DIV0, the cortical neurons were infected with LV.CMV.BDNF-mCherry for 24h. At DIV12,

neurons were imaged using an inverted microscope (Axio Observer, Zeiss) coupled to a spinning-disk confocal system (CSU-W1-T3, Yokogawa) connected to wide field electron-multiplying CCD camera (ProEM$^+$1024, Princeton Instrument) and maintained at 37 °C and 5% CO_2. We took images every 200 ms for 30 s to follow BDNF-mCh trafficking (×63 oil-immersion objective, 1.46 NA). Kymographs were generated using KymoToolBox plugin for ImageJ (57) with a length of 100 µm (x-axis) and a total time of 30 s (y-axis).

Antisense oligonucleotides

Antisense oligonucleotides (ASOs) used in this study are 2'-methoxyethyl modified ASOs with a mixed phosphorothioate/phosphodiester backbone (5'-CTCGACTAAAGCAGGATTTC-3') inducing mouse HTT pre-mRNA degradation. A scrambled sequence (5'- ACTTCGCAATACCGACTACA-3') was used as control obtained from Eurogentec. QRX-704 is a 2'O'methoxyethyl modified phosphorothioate ASO with the sequence 5'- GUCCCAUCAUUCAGGUCCAU -3' and was synthesized by LGC Bioresearch or ProQR Therapeutics.

Cell culture

Mouse Embryonic Fibroblasts (MEF) cultures were performed from E13.5 to E15.5 wt and *Htt*$^{\Delta 12/\Delta 12}$ knock-in mouse embryos. Fore and hind limbs of mouse embryos were dissected in PBS and digested 15 to 30 min at 37°C in trypsin-EDTA 0.05% with DNAse (Sigma-Aldrich, D5025, 250 U/ml). Trypsin was inactivated by addition v/v of MEF medium (1% Penicillin-Streptomycin (ThermoFisher Scientific, 15140122) and 10% of Fetal Bovine Serum (CVFSVF0001, Eurobio) in Dulbecco's Modified Eagle Medium (DMEM) GlutaMAX (ThermoFisher Scientific, 31966047). The pellet was recovered after 5 min of centrifugation at 300g and seeded in T75 flask in MEF medium. For Golgi reformation and ciliogenesis assays, fibroblasts were seeded in 12-well plates with 18 mm coverslips.

Cell transfection

For control experiments wt fibroblasts were transfected using lipofectamine 3000 (ThermoFisher Scientific, L3000015) with ASO or Scrambled (50nM). Lipofectamine 3000 quantities were used according to the manufacturer's instructions. Ciliogenesis was induced 24h after transfection and nocodazole treatment after 48h.

Western blotting

Cells were lysed in protein extraction buffer (Tris-HCl 20 mM, pH7.4, NaCl 137mM, EDTA 2mM, Triton X-100 1%, supplemented with 1/100 protease and phosphatases inhibitors (Sigma-Aldrich, P8340 and P5726)) after 48 hrs of transfection. Protein extracts were

denatured at 95°C for 5 minutes and then subjected to SDS-PAGE. Primary antibodies were used as follows: anti-HTT (Merck-Millipore #MAB2166, 1:1000), anti-vinculin (Sigma-Aldrich, V9131; 1:10 000).

Nocodazole treatment

Fibroblasts cells were treated with 4 μM nocodazole (Sigma-Aldrich, M1404) for 30 min at 4°C and 90 min at 37°C to allow a complete depolymerization of microtubules. Cells were washed twice with DMEM prior to methanol fixation (2 min at -20°C).

Ciliogenesis assays

For primary cilia induction, fibroblasts were shifted from 10% to 0.2% serum and fixed 48 hrs later with methanol (2 min at -20°C). For control experiments, ciliogenesis was induced 24 hrs after cells transfection with ASO or Scrb.

Immunofluorescence

After methanol fixation cells were incubated 1 hr at room temperature (RT) with blocking solution (0.1% Triton X100 and 5% bovine serum albumin in PBS-1X). The incubation with primary antibodies was made overnight at 4°C in blocking solution: Arl13b (UC Davis/NIH NeuroMab Facility, clone N295B, 1:500) and GM130 (BD Transduction Laboratories #610822, 1:500). After incubation 2h at RT with Alexa Fluor 555 mouse secondary antibody, samples were incubated with Hoechst (Sigma-Aldrich, B2261, 1:5000) and then coverslips mounted with Dako Fluorescent Mounting Medium (Agilent Technologies, S302380-2). Acquisitions were made using a 40X oil-immersion objective using an inverted confocal microscope (LSM 710, Zeiss). Cilia lengths were measured using ImageJ software and the size of Golgi particles were analyzed using analyses particles plugin of ImageJ software.

Animals

General: Mice were group housed (6/cage) prior to injection and solitary after injection under pathogen-free conditions with reversed light–dark cycle and cage enrichment in standard open polysulfone type II cages at constant temperature and humidity according to recommendations of the Federation of European Laboratory Animal Science Associations.

Tolerability, biodistribution and pharmacokinetics: Healthy CD1(ICR) or FVB/j mice were ordered from Charles River, France, both male and female (1:1 ratio), with an age range of 16-20 weeks (tolerability and biodisitribution) or 8-10 weeks (pharmacokinetics) equally divided over treatment groups at the moment of injection.

Dose-range in YAC128: Hemizygous FVB-Tg(YAC128)53Hay/J mice (YAC128) both male and female (1:1 ratio) with an age of at least 10 weeks at the start of the study will be used (equal age-range in treatment groups). Animals had been ordered from the Jackson Laboratory (58) and were further bred within the ProQR facilities, Leiden, The Netherlands. Mice genotyped by Transnetyx, Cordova, TN, USA by means of a standard PCR method on toes cut from newborn.
Phenotypic analysis in YAC128: Hemizygous FVB-Tg(YAC128)53Hay/J mice (YAC128) both male and female with an age of at least 8 weeks at the start of the study were used (equal age-range in treatment groups).

Intracerebroventricular (ICV) bolus injection

In short, for ICV bolus injections in the right lateral ventricle, mice were placed in a stereotaxic frame [Kopf] and anesthetized with 2% isoflurane/2%O_2 mixture by a nose cone fitted into the frame. The scalp and anterior back were shaved and disinfected. A 1–1.5 cm incision was made in the scalp, and the subcutaneous tissue and periosteum were scraped from the skull. A micro-drill was used to drill a hole through the skull at 0.22 mm posterior and 1.0 mm lateral to the bregma. Subsequently, a 10 µl Hamilton micro syringe with a 32 G type 2 point removable needle was lowered to a depth of 2.8 mm and a total volume of 10 µl test item was injected into the right lateral ventricle at a rate of 1 µl/s. After 3 min, the needle was slowly withdrawn and the incision sutured. Finally, the mice were allowed to recover from the anesthesia in their home cage (solitary).

ICV injection via cannula

A cannula was fixed to the skull of the mice to allow ICV bolus injections in the right lateral ventricle. In short, mice received an intraperitoneal (IP) injection of Buprenorphine (0.1 mg/kg) 15 min prior to the cannula placement. Subsequently, the mice were placed in a stereotaxic frame [Kopf] and anesthetized with 2% isoflurane/2%O_2 mixture by a nose cone fitted into the frame. The scalp and anterior back were shaved and disinfected. A 1–1.5 cm incision was made in the scalp, and the subcutaneous tissue and periosteum were scraped from the skull. A micro-drill was used to drill a hole through the skull at 0.22 mm posterior and 1.0 mm lateral to the bregma. A cannula was placed at a dept of 2.8 mm and fixed to the skull using dental cement; the skin was closed around the cannula using sutures. Finally, mice received a subcutaneous (SC) Carpofen (5 mg/kg) injection and were allowed to recover for a minimum of 14 days before administration of the first dose. ICV administration was performed while anesthetized with 2% isoflurane, protective cannula was unscrewed

and 5 µl (rate 1 µl/min) of test item was administered using a Hamilton syringe, with automatic injector, through the inner cannula into the lateral ventricle.

Necropsy and tissue collection

Mice were sacrificed using a mixture of 90 mg/kg ketamine and 12.5mg/kg/xylazine and subsequent transcardial perfusion with ice-cold HBSS via a peristaltic pump. Brains were carefully dissected and cut in half, separating the left and right hemispheres. For biochemical and molecular analysis, brains were further dissected into: (A) Cerebral cortex, (B) Cerebellum, (C) Hippocampus, (D) Midbrain and (E) Striatum. Tissues were immediately snap frozen in liquid N_2 and stored at -80℃. For histology, the right hemisphere was transferred to a tube containing MethaCarn, was fixed for 6 hrs, incubated in methanol for 1 hr and then processed to paraffin starting from xylene.

Fluorescent in-situ hybridization and GFAP immunostaining

Right brain hemispheres were fixated in MethaCarn (Methanol:Chloroform:Acetic acid/6:3:1) overnight and processed to paraffin starting from xylene. 10 µm sections were mounted on slides using a water bath. Slides were dried/baked overnight at 65°C before being dewaxed. After an absolute ethanol wash, the slides were air-dried before being heated for 15 min in Citrate 0.1M at 95°C for antigen retrieval, rinsed with demineralized water, and dried again. Pre-hybridization buffer (10 % formamide, 2x SSC (Sigma-Aldrich, S6639)), was added to the slides and incubated at 45°C for 30 min, after which hybridization solution (10% formamide, 2x SSC, 1 mg/ml yeast RNA (Sigma-Aldrich, R6750), 10 % dextran-sulfate (Sigma-Aldrich, D8906), 25 nM Cy5-conjugated LNA/DNA mixer probe (Eurogentec)) was incubated at 45°C for 3 hrs. The sections were washed 3 times with 45°C hybrid-wash (10 % formamide, 0.1x SSC) before being rinsed in PBS with 0.1 % Tween20. For anti-GFAP immunostaining, sections were blocked with 10% BSA in PBS, and incubated overnight at 2-8°C with an anti-GFAP antibody (Dako #Z0334). Upon washing with PBS with 0.1% Tween20, slides were incubated with an Alex568-conjugated goat-anti-rabbit secondary antibody (ThermoFisher Scientitifc, #A11011) for 2 hrs at RT, followed by washing with PBS with 0.1% Tween-20. Sections were mounted with a DAPI containing mounting medium.

Bioanalysis

Bioanalysis was performed by QPS Netherlands using hybridization ELISA by capturing QRX-704 with a biotinylated oligonucleotide capture probe complementary to the 3' end of QRX-704, onto a streptavidin-coated MSD Plate (Meso Scale Diagnostics), and detected with a digoxigenin-labeled detection oligonucleotide probe complementary to the 5' end of

QRX-704, using the Meso Scale detection system (Meso Scale Diagnostics) as described previously (59).

Porsolt's forced swim test (FST)

Mice were recorded during a 6-min forced swim in a 25 cm × 19 cm cylinder filled with 25-30°C water. A fully automated video tracking system was used: EthoVision® XT (Noldus) to distinguish movement and immobility. The time spent immobile (floating) during the final 4 min was used as outcome measure.

Rotarod

Rotarod testing was performed using an accelerating rotarod apparatus (UGO Basile, Comerio, Italy). After three consecutive days training with constant speed, three trials were performed spaced 2 hrs apart using an accelerating rotarod, from 5 to 40 RPM over 5 min, allowing a maximum score of 300 s. Latency to fall was defined by either the animal falling from the rotarod or when the mouse could no longer remain upright and rotated over 180°. The latency to fall was recorded for each of the three trials to calculate the mean latency to fall per mouse. Animals were tested monthly (on 1 day with 3 trials) from 2 months to 13 months age.

YAC128 phenotype study western blot

Protein was extracted with T-PER buffer (ThermoFisher Scientific, 87510) from dissected whole mouse brains by homogenization with the Dounce tissue grinder set (Sigma-Aldrich, D8938). Protein concentrations were measured by BCA assay (Pierce®) according to manufacturer's instructions. Criterion XT Precast Gel 3-8% (Biorad) for detection of Δ12HTT and 10% Bis-Tris for detection of HTT 586 N-terminal fragments from BioRad were used to load 40 µg and 100 µg of protein, subsequently, in 4x NuPAGE LDS Sample buffer (ThermoFisher Scientific, lot#: 1386570) after sample was denatured by heating to 95˚C. Tricine (BioRad) running buffer was used to ran gels at 100 V constant for ~ 5hrs. Proteins were transferred to an Immobilon-nitrocellulose 0.2 µm membrane and probed with the following antibodies: primary Huntingtin (D7F7) XP® Rabbit mAb (Cell Signaling Technology®, #5656, 1:2000); primary Huntingtin Δ12 (26E7) Mouse mAb (in-house, 1:1000); primary anti-polyglutamine-expansion diseases marker Mouse monoclonal antibody, clone 5TF1-1C2 (Merck, MAB1574, 1:2000); primary anti-Vinculin Rabbit monoclonal antibody [EPR8185] (Abcam, ab129002) as a loading control; secondary 800 CW goat anti-mouse (LI-COR Biosciences®, 1:10000); secondary 680 RDye goat anti-rabbit (LI-COR Biosciences®, 1:10000).

YAC128 phenotype study immunohistochemistry

Right hemispheres were extracted from sacrificed YAC128 mice and formalin fixed at 2-8°C. Brain tissue was then immersed in 30% sucrose at RT for 2 days or until they had sunken to be subsequently cryo-frozen in a sample block using Tissue-Tek O.C.T.™ compound (Saikura, cat#4583). Sagittal cryo-sectioning was performed, and slides were stored at -20˚C until immunostaining. 20 min antigen retrieval in 0.01M sodium citrate buffer, pH 6, at 95˚C was followed by a water rinsing and drying step at RT. Sections were blocked in 3% Normal Goat Serum (NGS)-0.1% Triton-x100/PBS for 2 hours at RT to subsequently be incubated with primary antibodies for EM48 (Sigma-Aldrich, MAB5374, 1:500) and Spinophilin (Merck, 06-852, 1:250) in 3% NGS-0.1% Triton X-100-PBS (PBS-NT buffer) at 4˚C O/N. After extensive washing with PBS/0.1%Tween20 buffer (Gibco,18912-014; Sigma-Aldrich, P1379), secondary antibodies GxM^{647} (EM48, 1:500, Abcam, Ab150115) and GxR^{568} (ThermoFisher Scientific, A11011, 1:500) were diluted in PBS-NT with 1:1000 Hoechst 33342 (ThermoFisher Scientific, H3570) and applied for 2h at RT. Upon extensive washing in PBS/Tween20, sections were mounted in AquaPolymount (Tebubio,18606-20), left to dry and stored at 4˚C until imaging. Imaging was performed on a Zeiss LSM800 scanning confocal microscope equipped with airyscan. 5 Z-stack images per location in cortex and striatum were recorded using the Plan Apochromat/1.3 Oil 40x objective. (LSM800 Airyscan, 40x1.3, Z-stack 5x0.3µm, 1232x1232 pixels, Channels(ex-em): Hoechst 348-455nm / AF^{568} 577-608nm / AF^{647} 658-668nm, Filters: Hoechst 400-520nm, Alexa Fluor^{568}: 500-633nm, Alexa Fluor^{647}: 645-700nm, pixeltime 3,43µs). ImageJ was used for the image analysis using the cell counter tool for the blinded manual counting of EM48-positive cells, and the threshold and analyse particles tool to define the number of spinophilin puncta per image. GraphPad Prism was used to perform statistics employing an ordinary one-way ANOVA test and Tukey test.

***In vitro* caspase-6 cleavage assay and western blot**

To track the presence of 586 N-terminal fragments in protein samples prepared from Bl/6 mouse brains (isolated as described above), 40 µg of protein sample were mock-treated or incubated with recombinant Caspase6 (human recombinant Caspase6 100 Units, lot#: 2992850, Millipore) O/N at 37˚C. 4x NuPAGE LDS Sample Buffer (ThermoFisher Scientific lot#: 1386570) and 50 mM DTT were added to the samples, that were subsequently boiled at 95˚C for 5 min. A 10% Criterion™ XT Bis-Tris Protein Gel (BioRad, cat#: 3450111) and 1x XT MOPS Running Buffer (BioRad, cat#: 1610788) were used for Western blot analysis of the described samples. Gels were run for ~ 5hrs at a constant 100 V and proteins were transferred to an Immobilon-nitrocellulose 0.2 µm membrane. The latter was incubated with

the following antibodies: primary mouse-anti-HTT (MAB2166 4C8, 1:2.000) and secondary 800 CW goat anti-mouse (LI-COR Biosciences®, 1:10.000).

COS7 caspase-6 assay

COS7 cells were transfected with HTT wt or HTTΔ12 plasmids using Lipofectamine 2000 (ThermoFisher Scientific, 11669019) for 24 hrs. Cells were collected in caspase assay buffer containing 50 mM Hepes, 50 mM NaCl, 10 mM EDTA and 0.1% 3-[(3-cholamidopropyl)dimethylammonio]-1-propanesulfonate (CHAPS). Protein concentrations were determined using the BCA assay as described above, 10 mM DTT was added to the lysates, and reactions containing 5 µg total protein were setup with 0-4.0 units of Caspase-6 (Biovision Cat no 1086-100), incubated at 37˚C for 5 hrs.

Statistical analyses

GraphPad Prism (GraphPad Software, Inc.) software was used for statistical analysis. All experiments consisted of at least three independent replicates (Figure 4, 5 and 6). For the transport data: we only performed two independent cultures but compared 84 $Htt^{\Delta12/\Delta12}$ axons and 84 wt axons coming from five different embryos for each genotype. D'Agostino & Pearson normality test two groups were compared using the unpaired two-tailed Student's test or Mann-Whitney test. Data are expressed as mean +/- S.E.M. Ns: no significant; * $p < 0.05$; ** $p<0.01$.

Study approval

Mouse experiments were approved by the local Ethics Committee and conformed to the European Community regulations (CEE n° 86/609)
